# Neutrophils promote the activation of monocytes via ROS to boost systemic antitumor immunity after cryo-thermal therapy

**DOI:** 10.3389/fimmu.2024.1445513

**Published:** 2024-11-01

**Authors:** Shicheng Wang, Zelu Zhang, Junjun Wang, Yue Lou, Yongxin Zhu, Jiaqi You, Ping Liu, Lisa X. Xu

**Affiliations:** School of Biomedical Engineering and Med-X Research Institute, Shanghai Jiao Tong University, Shanghai, China

**Keywords:** neutrophil, monocyte, ROS, cryo-thermal therapy, antitumor immunity

## Abstract

**Background:**

The characteristics of the tumor immunosuppressive microenvironment represent a major challenge that limits the efficacy of immunotherapy. Our previous results suggested that cryo-thermal therapy, a tumor ablation system developed in our laboratory, promotes macrophage M1-type polarization and the complete maturation of DCs to remodel the immunosuppressive environment. However, the cells that respond promptly to CTT have not yet been identified. CTT can cause extensive cell death and the release of danger-associated molecular patterns and antigens. Neutrophils are the first white blood cells recruited to sites of damage and acute inflammation. Therefore, we hypothesized that neutrophils are the initial cells that respond to CTT and are involved in the subsequent establishment of antitumor immunity.

**Methods:**

In this study, we examined the kinetics of neutrophil recruitment after CTT via flow cytometry and immunofluorescence staining and explored the effect of neutrophils on the establishment of systemic antitumor immunity by *in vivo* neutrophil depletion and *in vitro* co-culture assays.

**Results:**

We found that CTT led to a rapid and strong proinflammatory neutrophil response, which was essential for the long-term survival of mice. CTT-induced neutrophils promoted the activation of monocytes via reactive oxygen species and further upregulated the expression of IFN-γ and cytotoxic molecules in T and NK cells. Adoptive neutrophil transfer further enhanced the antitumor efficacy of CTT in tumor models of spontaneous and experimental metastasis.

**Conclusion:**

These results reveal the important role of neutrophil‒monocyte interactions in the development of anti-tumor immunity and highlight that CTT could be used as an immunotherapy for targeting neutrophils and monocytes to enhance antitumor immunity.

## 1 Introduction

Tumor immunotherapies including immune checkpoint blockade and adoptive T-cell transfer therapy have been used to treat many different types of advanced cancer due to their durable and robust effects on various malignant tumors (1). Despite the tremendous successes of immunotherapy, the successes to date do not fully reflect the promise of immunotherapy (1) because only 30% of the patients experience an objective response (2). The ability of the tumor microenvironment to orchestrate immune tolerance and T-cell exclusion is a major challenge that limits the efficacy of immunotherapy (3). However, current immunotherapies focus on mobilizing the adaptive compartment of the immune system, and the role of the innate immune system in tumor immunotherapies is ignored in many studies.

In our previous study, we developed a cryo-thermal therapy (CTT) that could ablate tumors locally and effectively destroy tumor cells by combining precooling and subsequent radiofrequency ablation. CTT effectively remodeled the immune environment, with macrophages polarized toward the M1 phenotype, eosinophils activated on day 5 post treatment and DCs fully matured on day 14 post treatment (4, 5). However, the immune cells that respond first in the early stage after CTT have not been identified.

Neutrophils are the most prevalent type of immune cell with the highest proportion of circulating myeloid cells (6). They have long been regarded as short-lived effector cells with limited capacity for biosynthetic activity and they play a major role in resisting extracellular pathogens and acute inflammation (7). However, this framework has been challenged by the demonstration that neutrophils survive much longer than previously thought and can produce several key cytokines and chemokines (3, 7). In the context of cancer, cytokines produced by tumor cells and stromal cells develop the immunosuppressive characteristics of neutrophils (8). In turn, neutrophils can promote tumor progression and inhibit the antitumor response (9). Nonetheless, there is growing evidence to support the idea that neutrophils exert an antitumor effect under certain conditions. Neutrophils can directly kill tumor cells in a ROS, elane, or iNOS-dependent manner (10–13), or exert antitumor effects by activating other immune cells including T-cells and NK cells (14–17). Therefore, new tumor immunotherapy strategies that enable protumor neutrophils to exert their potential antitumor functions are needed.

Neutrophils are the first cells recruited to sites of inflammation and rapidly accumulate in damaged tissue (6). In a heat-induced sterile inflammation model, neutrophils were found to adhere to the vascular endothelium around the injured area 30–60 min after injury and were recruited to the necrotic tissue for further phagocytosis and clearance of tissue debris (18, 19). Due to its ability to induce tumor cell necrosis, CTT promotes the release of large amounts of danger-associated molecular patterns (DAMPs) from tumor sites (20, 21). Because neutrophils are the first cells to be recruited to injury sites, we hypothesized that neutrophils are the first cells to respond to CTT and are involved in CTT-induced systemic antitumor immunity.

In this study, we investigated the dynamic changes and the role of neutrophils in CTT-induced systemic antitumor immunity. We found that CTT induced a rapid neutrophil response, which was accompanied by the production of high levels of reactive oxygen species (ROS), which in turn promote the activation of monocytes. Neutrophil-activated monocytes further promoted the Th1 differentiation of CD4^+^ T-cells and the effector function of CD8^+^ T-cells and NK cells, thus triggering optimal systemic antitumor immunity, which is necessary for the long-term survival of mice. CTT combined with adoptive neutrophil transfer further contributed to the inhibition of metastatic tumors. The results of this study reveal a novel mechanism by which neutrophil and monocyte interactions orchestrate systemic antitumor immunity.

## 2 Materials and methods

### 2.1 Animal model

Female C57BL/6 mice and BalB/c mice (Shanghai Slaccas Experimental Animal Co., Ltd, China) were housed and fed sterile food with standard mice nutritional formula and sterile water in the isolated cages of 12 h light/dark cycle environment. B16F10 cells, MC38 cells and 4T1 cells were cultured in DMEM medium (Hyclone, USA) supplemented with 10% fetal bovine serum (FBS, Gemini Bio-Products, West Sacramento, California, USA) and penicillin-streptomycin (Hyclone). To prepare the tumor-bearing mice, B16F10 cells (5×10^5^) and 4T1 cells (4×10^5^) were injected subcutaneously (s.c.) into right flank of mice when the mice were 6-8 weeks old and weighed 20 g. To prepare the MC38 bilateral tumor-bearing mice, 5×10^5^ and 1×10^5^ MC38 cells were injected subcutaneously (s.c.) into right flank and left flank of mice respectively. Tumor-bearing mice were then randomly assigned to housing cages, and subjected to different treatments. All animal experiments were approved by the Animal Welfare Committee of Shanghai Jiao Tong University and experimental methods were performed in accordance with the guidelines of Shanghai Jiao Tong University Animal Care (approved by Shanghai Jiao Tong University Scientific Ethics Committee, Registration No. 2020017).

### 2.2 The treatment procedures

Cryo-thermal therapy was performed when the average tumor size reached about 0.2 cm^3^. Briefly, the subcutaneous tumor of mice in cryo-thermal therapy group was frozen with liquid nitrogen to -20°C for 5 minutes, then heated with radiofrequency to 50°C for 10 minutes.

For CTT with adoptive neutrophil transfer, neutrophil was isolated from bone marrow of naïve mice using Mouse Neutrophil Enrichment Kit (STEMCELL Technologies, Canada) according to the instructions. Mice were adoptively transferred with neutrophils i.v. 15 min before CTT.

For ROS clearance *in vivo*, mice treated with CTT were administered L-Glutathione reduced (GSH, 10 mg/kg, Yeasen, China) intraperitoneally twice daily for a total of three days.

### 2.3 Single-cell suspension preparation and flow cytometry analysis

The spleens, blood, lungs and tumors were collected after therapy. A single-cell suspension of splenocytes was prepared using GentleMACS dissociator (Miltenyi Biotec, Germany). The tumors and lungs were digested with collagenase I (Yeasen), hyaluronidase (Yeasen, China) and DNase I (Yeasen, China) and the tissues were mashed through a 70 mm cell strainer (Falcon, USA). Red blood cells were removed by erythrocyte-lysing reagent containing 0.15 M NH_4_Cl, 1.0 M KHCO_3_, and 0.1 mM Na_2_EDTA. Zombie Aqua™ Fixable Viability Kit (BioLegend, USA) was used to assess the cell viability. CD16/CD32 antibody (Bio-X-cell, USA) was used for Fc receptor blocking. Precision Count Beads (BioLegend, USA) were used to obtain absolute counts of cells. Staining antibodies were described at **Supplementary Table 1**. For cell surface staining, cells were stained with antibodies for 30 min at 4 °C. For intracellular staining, cells were stimulated for 4 h with Cell Activation Cocktail (phorbol-12-myristate 13-acetate, ionomycin, and Brefeldin A, Biolegend, USA) according to the manufacturer’s protocol. Briefly, the cells were surface stained with antibodies binding cell-specific surface marker and fixed and permeabilized using Fixative Buffer (Biolegend) and Intracellular Staining Perm Wash Buffer (Biolegend, USA), respectively. Subsequently, the cells were incubated with antibodies specific for cytokines for 30 min at 4°C. True-Nuclear™ Transcription Factor Buffer Set (Biolegend, USA) was used to analyze the expression of transcription factors. The cells were stained with a surface antibody and subsequently fixed for 45 min using Fix Concentrate. Following this, the cells were washed three times with Perm Buffer. Subsequently, transcription factor antibodies were added and incubated for a further 60 min at 4°C. The level of ROS was measured by DCFH-DA (Beyotime Biotechnology, China) for 15 min at 37°C. Cell fluorescence was assessed with a FACS AriaII or Fortessa (BD Biosciences, USA) and analyzed with FlowJo software (version 10.6.2).

### 2.4 Immunofluorescence staining and immunohistochemical staining

For immunofluorescence staining, slides were cut to 10 μm and blocked with 2% Bovine Serum Albumin (Yeasen, China) for 1 hour. APC anti-Ly6G was used for immunofluorescence. Images were acquired by quantitative laser scanning confocal microscopy (Leica TCS SP5, Germany). For immunohistochemical staining, heat-induced antigen retrieval was preformed using sodium citrate buffer. Then endogenous peroxidase was removed using Endogenous Peroxidase Blocking Buffer (Beyotime Biotechnology, China). Slides were blocked with 2% Bovine Serum Albumin for 1 hour. Purified anti-Ly6G (clone 1A8, Bio-X-Cell, USA) and HRP anti-rat IgG (Beyotime Biotechnology, China) were used for immunohistochemical staining. Then the slides were stained with diaminobenzidine (DAB) kit (Beyotime Biotechnology, China) at room temperature for 10 min in the dark, followed by counterstaining with hematoxylin for cell nuclei. Images were acquired with a Leica microscope (Leica DM6 B, Germany).

### 2.5 Depletion of neutrophil and monocyte

For neutrophil depletion, 100 μg of anti-Ly6G (Bio-X-cell, USA) was i.p. injected 12 h before CTT. For monocyte depletion, 200 μL of clodronate liposomes or phosphate-buffered saline (PBS) liposomes (Liposoma BV, Netherlands) were injected i.v. on day 5 after CTT. The efficiencies of neutrophil and monocyte depletion were assessed by flow cytometry assay.

### 2.6 Isolation of neutrophils and monocytes

For RNAseq, neutrophils and monocytes were labeled with PE anti-Ly6G and PE anti-CCR2 and isolated from spleen by EasySep Mouse PE Positive Selection Kit II (STEMCELL Technologies, Canada) according to the instructions. In order to study neutrophil function, the EasySep Mouse Neutrophil Enrichment Kit (STEMCELL Technologies, Canada) was used for the isolation of neutrophils.

### 2.7 Obtaining conditioned medium for neutrophils and *in vitro* cell co-culture

Neutrophils from untreated mice or CTT mice were sorted by MACS and cultured for 24 hours. The culture supernatant was subjected to centrifugation at 400 g for 10 minutes, followed by centrifugation at 2000 g for an additional 10 minutes, thereby obtaining neutrophil-conditioned medium. Neutrophil-free splenocytes or monocytes were cocultured with neutrophils or neutrophil-conditioned medium in the presence of GSH (MedChemExpress, China) at concentrations of 5 mM and 10 mM. To investigate the direct effect of ROS on MHC-II expression in monocytes, monocytes were treated with H_2_O_2_ at concentrations of 10 µM, 50 µM and 100 µM for 24 h.

### 2.8 *In vitro* killing assay

Cell killing capacity was assessed using the calcein release assay (22). In brief, CD4^+^T-cells, CD8^+^ T-cells and NK cells were co-incubated with Calcein-AM-loaded B16F10 cells for 6 h at a ratio of 8:1, 4:1, 2:1 and 1:1. Subsequently calcein fluorescence in the supernatant was detected using SpectraMax i3x (Molecular Devices, USA).

### 2.9 RNA-seq and analysis

Total RNA was extracted using the TRIzol reagent (Invitrogen, CA, USA) and the purity and quantification of RNA were evaluated using the NanoDrop 2000 spectrophotometer (Thermo Scientific, USA). RNA integrity was assessed by using the Agilent 2100 Bioanalyzer (Agilent Technologies, Santa Clara, CA, USA). Then the libraries were constructed using VAHTS Universal V6 RNA-seq Library Prep Kit. The transcriptome sequencing and analysis were conducted by OE Biotech Co., Ltd. (Shanghai, China). PCA analysis was performed using R (v 3.2.0) to evaluate the biological duplication of samples. Gene Set Enrichment Analysis (GSEA) was performed using GSEA software (23). Bioinformatic analysis was performed using the OECloud tools at https://cloud.oebiotech.com.

### 2.10 Statistical analysis

The results were expressed as the mean ± standard deviation (SD) unless otherwise specified. Statistical analyses were conducted with One-way ANOVA-test (for multiple groups comparisons) or Student’s t-test (for comparisons between two groups). Log rank test was used for the survival analysis. Statistical significance was defined as p values less than 0.05: *p < 0.05, **p < 0.01, ***p < 0.001, and ****p < 0.0001. The statistical analyses were performed using GraphPad Prism 10 software. Exact n values and the number of independent experiments are provided in the figure legends.

## 3 Results

### 3.1 Cryo-thermal therapy (CTT) induced a systemic neutrophil response at an early stage

**Figure 1:**
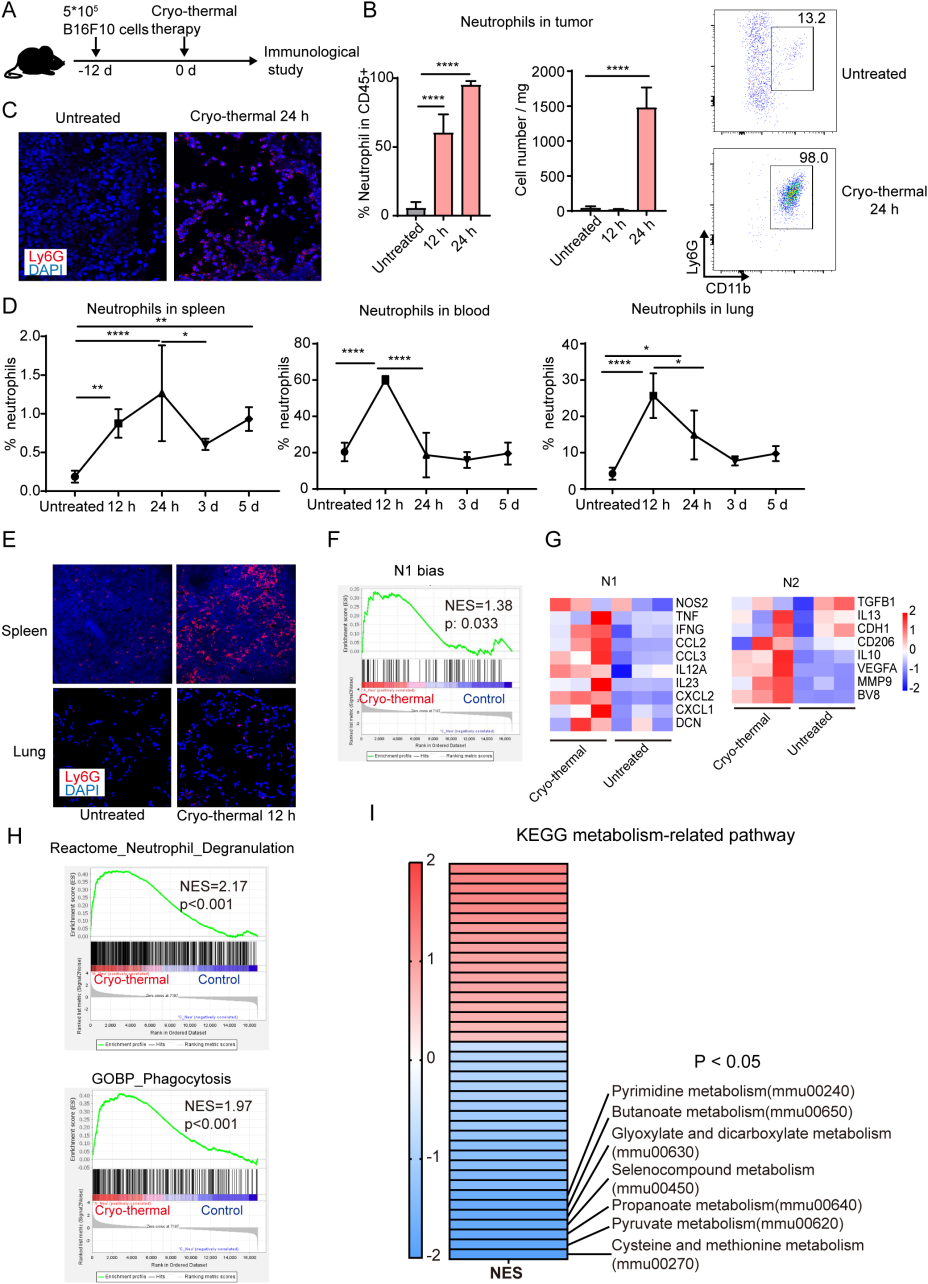
Neutrophils induced by CTT were essential for the long-term survival of mice. **(A)** Scheme of experiment design. **(B)** The percentage and absolute count of neutrophils in tumor were measured by flow cytometry. **(C)** Representative immunofluorescence staining images of neutrophil (Ly6G^+^, red) infiltration in tumor of tumor-bearing mice and CTT mice at 24 h after CTT in the B16F10 tumor models. **(D)** The percentage of neutrophils in spleen, blood and lung 12 h, 24 h, 3 d and 5 d after CTT was detected by flow cytometry in the B16F10 tumor models. **(E)** Representative immunofluorescence staining images of neutrophil (Ly6G^+^, red) infiltration in spleen and lung of tumor-bearing mice and mice at 12 h after CTT. **(F)** Neutrophil from untreated mice or CTT mice using B16F10 tumor model was sorted for RNAseq and genes associated with the set of genes previously implicated in the TAN1 phenotype were analyzed using GSEA. **(G)** N1 and N2 associated genes were upregulated after CTT as compared to untreated mice. **(H)** The gene sets of neutrophil degranulation pathway (from Reactome Pathway Database) and phagocytosis pathway (from GO Database) was analyzed by GESA. **(I)** Heatmap showing the NES of GSEA based on the KEGG metabolism-related pathway gene set. *p <0.05, **p <0.01, ****p <0.0001, n=4 for flow cytometry and n=3 for RNA-seq.

Neutrophils are the first cells to reach an injured site by sensing DAMPs released by necrotic cells (18, 19). The results of our previous studies revealed that CTT induced tumor cell necrosis and released large amounts of DAMPs (20, 21). However, the recruitment dynamics of neutrophils after CTT remain unclear. Therefore, the changes in neutrophils in the B16F10 tumor models were studied at different time points after CTT (**Figure 1A**). Compared with those in the untreated group, the absolute numbers of neutrophils in the tumors were unchanged at 12 h after CTT but were dramatically increased at 24 h after CTT (**Figure 1B**). A significant increase in the proportion of neutrophils within the tumor was observed at both 12 and 24 hours after CTT (**Figure 1B**). At 24 h after CTT, the majority of immune cells observed within the tumor mass were neutrophils, representing a proportion exceeding 90%. (**Figure 1B**). Immunofluorescence staining also showed that many Ly6G^+^ neutrophils infiltrated into the tumors at 24 h after CTT (**Figure 1C**). Moreover, the proportions of neutrophils in the spleen, blood and lungs were significantly increased at 12 h after CTT (**Figure 1D**). Immunofluorescence staining further demonstrated that neutrophils accumulated in the spleen and lung 12 h after CTT (**Figure 1E**). At 24 h after CTT, the proportion of neutrophils in the spleen continued to increase, but decreased in the blood and lungs, and remained at the baseline level at 3 d and 5 d after CTT (**Figure 1D**). Although the percentage of neutrophils in the spleen was slightly reduced at 3 and 5 d after CTT, their proportion was still higher than that in untreated mice (**Figure 1D**). The proportion of other immune cells was not found to be elevated after CTT (**Supplementary Figure 1**). In the 4T1 breast cancer models, although the baseline levels of neutrophils in blood and lungs reached approximately 80%, the proportions of neutrophils in the tumor, blood and lung increased at 12 hours after CTT and decreased at 24 h and 3d after CTT (**Supplementary Figure 2**). These results suggest that CTT induced a rapid neutrophil response.

To further analyze the active status of neutrophils, splenic neutrophils were sorted for RNAseq. Gene set enrichment analysis (GSEA) using a gene set associated with the N1 phenotype indicated that neutrophils were significantly polarized toward the N1 phenotype after CTT compared to those of untreated mice (**Figure 1F**). Compared with that in untreated mice, genes associated with the pro-inflammatory phenotype of N1 neutrophils were upregulated in mice that received CTT, while the expression of genes associated with anti-inflammation and angiogenesis was simultaneously upregulated (**Figure 1G**). Neutrophil degranulation and phagocytosis pathways associated with neutrophil activation were significantly enriched after CTT (**Figure 1H**). In addition, the changes in the metabolic pathways of neutrophils were analyzed after CTT. The results showed that cysteine and methionine metabolism was the most significantly down-regulated pathway (**Figure 1I**). MDSCs can inhibit the function of T cells by competing for cysteine (24), so the down-regulation of cysteine and methionine metabolism indicates reduced immunosuppression of neutrophils after CTT. These results suggest that CTT-induced neutrophils were markedly activated.

### 3.2 CTT-activated neutrophils promoted monocyte activation via ROS

**Figure 2:**
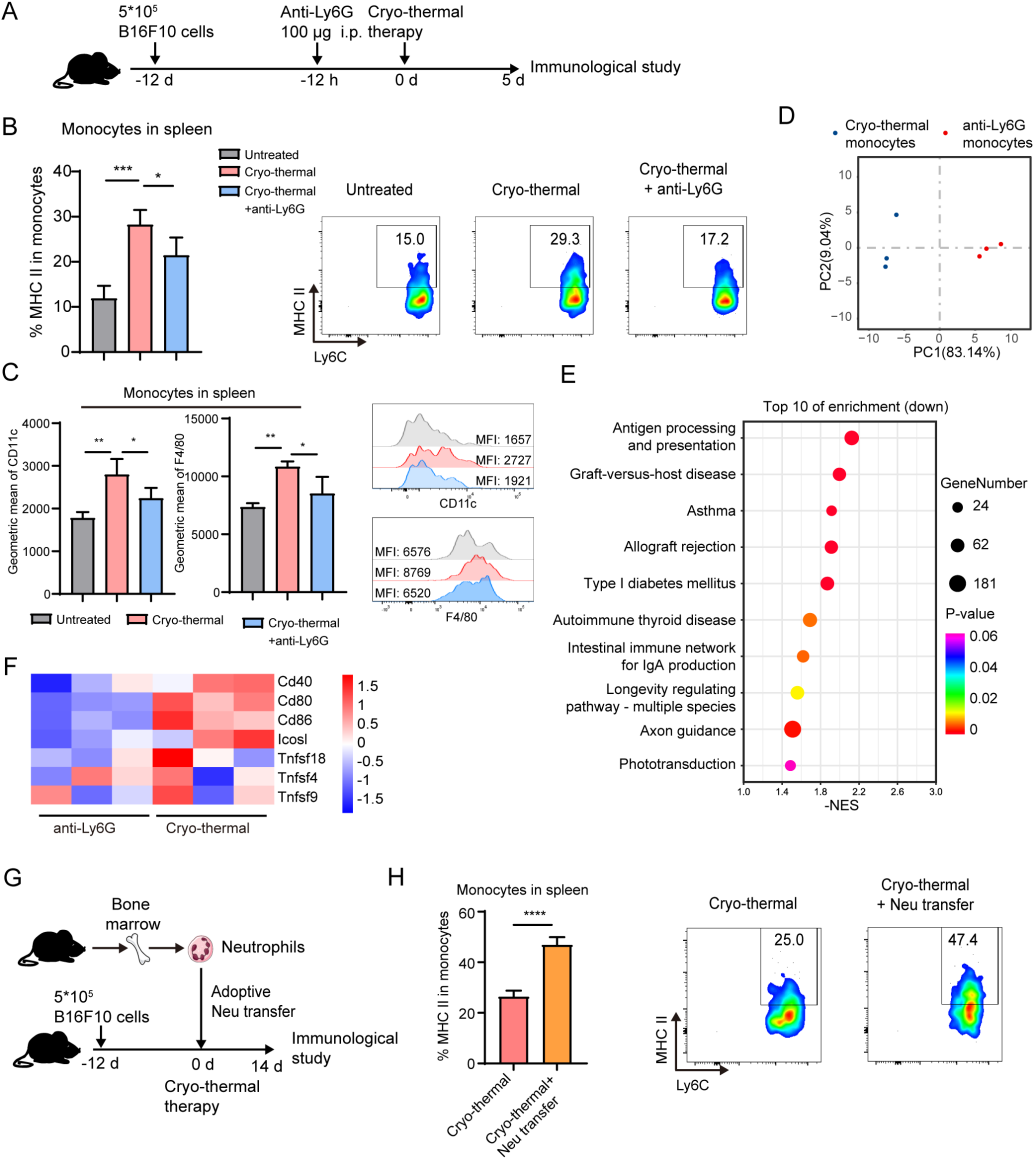
Neutrophils induced by CTT promoted the activation of monocytes. **(A)** Scheme of experiment design. **(B, C)** The expression of MHC-II, CD11c and F4/80 in monocyte in spleen on day 5 after CTT was measured by flow cytometry. **(D–F)** Monocytes from CTT and anti-Ly6G group was sorted by MACS on day 5 after CTT and analyzed by RNA-seq. **(D)** PCA analysis were performed to evaluate the biological duplication of monocytes. **(E)** Top 10 enriched gene sets analyzed by GESA based on KEGG terms. **(F)** Heatmaps showing expression levels of co-stimulatory molecule-related genes on mouse spleen monocytes in the CTT and CTT with anti-Ly6G groups. **(G, H)** B16F10 tumor-bearing mice were adoptively transferred with neutrophils from bone marrow of naïve mice 15 min before CTT and the expression level of MHC-II on splenic monocytes was detected on day 14 after CTT. *p <0.05, **p <0.01, ***p <0.001,****p<0.0001. n=4 for flow cytometry and n=3 for RNA-seq.

To further investigate the role of the systemic neutrophil response at an early stage in the antitumor activity induced by CTT, anti-Ly6G antibody was administered 12 h before CTT to deplete the neutrophils and the changes in other immune cells were analyzed at 5 d after CTT (**Figure 2A**). The gating strategy is shown in **Supplementary Figure 3**. Flow cytometry analysis showed a significant reduction in the proportion of circulating neutrophils from approximately 20% to 4.2% after CTT with anti-Ly6G antibody treatment compared with that in the untreated mice and CTT-treated mice (**Supplementary Figure 4A**). Compared with that in the untreated group, the expression of MHC-II on monocytes from the spleen and lung markedly increased after CTT (**Figure 2B**, **Supplementary Figure 4B**). The expression of MHC-II on monocytes from the blood of CTT-treated and untreated mice was comparable (**Supplementary Figure 4B**). Monocytes can further differentiate into monocyte derived DCs and monocyte derived macrophages after stimulation (25). Compared with those in the untreated group, the expression of CD11c (a DC-specific marker) and F4/80 (a macrophage-specific marker) was significantly increased in the spleen but not in blood and lung after CTT (**Figure 2C**). Interestingly, after CTT with anti-Ly6G treatment, the levels of MHC-II, CD11c and F4/80 on splenic monocytes were decreased compared with those in the CTT group (**Figure 2B**), although the activation of monocytes in the blood and lungs was not markedly changed (**Figure 2B**, **Supplementary Figure 4B**). These results indicate that the CTT induced neutrophil response at an early stage significantly promoted the activation of monocytes.

Although the expression of MHC-II on macrophages and DCs did not markedly change in the spleen or lung after CTT compared with that in the untreated group, neutrophil depletion led to a slight decrease in the expression of MHC-II on splenic DCs, and macrophages from the spleen and lung after CTT (**Supplementary Figure 4C**). To further explore the ability of neutrophils to regulate the function of lymphocytes at an early stage after CTT, IFN-γ and perforin in T cells and NK cells were measured by flow cytometry. Compared with those in the CTT group, anti-Ly6G treatment did not change the levels of IFN-γ and perforin in CD8^+^ T cells and NK cells after CTT (**Supplementary Figures 5A, B**). Moreover, the expression of IFN-γ was increased and the expression of perforin was decreased in CD4^+^ T-cells after CTT with anti-Ly6G treatment compared with those in the CTT group (**Supplementary Figures 5A, B**). Overall, these results suggest that CTT-induced neutrophils affect mainly monocytes and promote their activation, although CTT also has a slight effect on CD4^+^ T-cell function.

To further investigate the role of neutrophils in the maturation of monocytes, splenic monocytes from CTT and CTT with anti-Ly6G treated mice were sorted and RNA-seq was performed. Two-dimensional principal component analysis (PCA) and cluster analysis showed that monocytes from the CTT with anti-Ly6G group expressed a gene expression profile distinct from that of CTT group (**Figure 2D**). To explore the alterations in pathways associated with the immune response after CTT with neutrophil depletion, gene set enrichment analysis (GSEA) was conducted. Based on the KEGG terms, GSEA revealed that the antigen processing and presentation pathway (mmu04612) was the most significantly downregulated pathway in monocytes (**Figure 2E**). Meanwhile, the peptide antigen binding (GO:0042605) and T-cell receptor binding (GO:0042608) pathways were downregulated in monocytes after CTT with neutrophil depletion compared with those from CTT-treated mice (**Supplementary Figures 6A, B**). Moreover, the expression of *CD80*, *CD86*, *CD40* and *ICOSL* on monocytes was comprehensively downregulated after CTT with neutrophil depletion compared with CTT alone (**Figure 2F**). These data reveal that CTT with anti-Ly6G treatment substantially impaired the capacity of antigen processing and presentation in monocytes induced by neutrophils. To further validate the activating effect of neutrophils on monocytes, neutrophils from the bone marrow of naïve mice were adoptively transferred prior to CTT to amplify the neutrophil response (**Figure 2G**). Compared with that of CTT alone, the expression of MHC-II in monocytes was further increased after the adoptive transfer of neutrophils (**Figure 2H**). These results strongly imply that the neutrophil response after CTT promoted the activation of monocytes.

**Figure 3:**
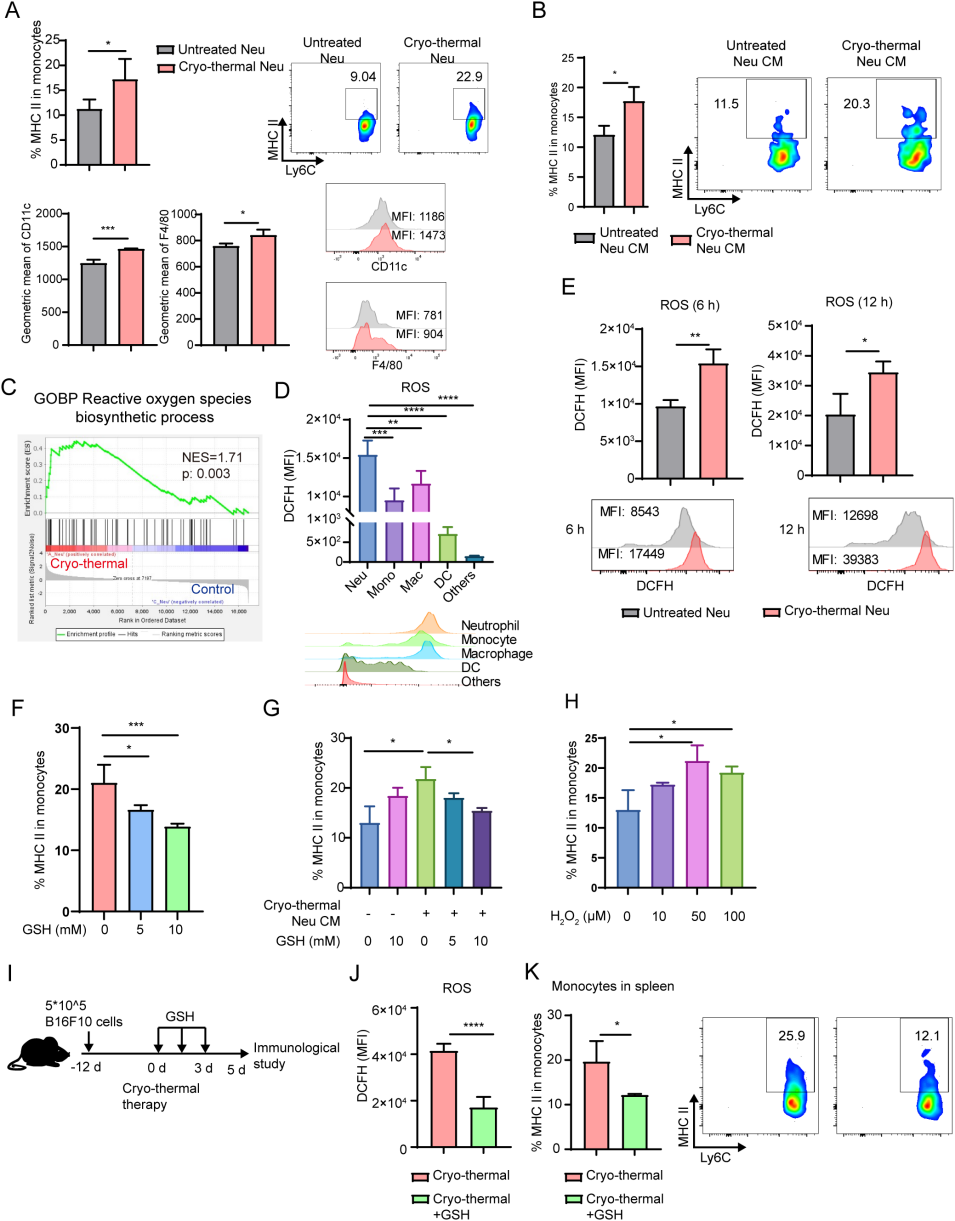
Neutrophil promoted the maturation of monocyte by ROS. **(A)** Neutrophils sorted from untreated mice and CTT mice in B16F10 tumor models were cocultured with neutrophil-free splenocytes at a ratio of 1:5 for 24h. The expression of MHC-II, CD11c and F4/80 on monocytes was detected by flow cytometry. **(B)** Neutrophils from untreated mice or CTT mice were sorted by MACS and cultured for 24 hours to obtain neutrophil-conditioned medium (CM). Monocytes were treated with different conditioned medium for 24 h and the expression of MHC-II on monocytes was measured by flow cytometry. **(C)** The ROS biosynthetic process pathway was analyzed by GESA. **(D)** The level of ROS in neutrophils, monocytes, macrophages, DCs and other cells was matured by DCFH-DA using flow cytometry. **(E)** The level of ROS in splenic neutrophils at 6 h and 12 h after CTT was matured by DCFH-DA using flow cytometry. **(F)** Splenic neutrophils and monocytes were sorted and cocultured for 24 hours in the absence or presence of 5 mM and 10 mM GSH. The expression of MHC-II was detected by flow cytometry. **(G)** Monocytes were treated with different conditioned medium with or without GSH for 24 h and the expression of MHC-II on monocytes was measured by flow cytometry. n=3. **(H)** Monocytes were treated with H_2_O_2_ at concentrations of 10 µM, 50 µM and 100 µM for 24 h and the expression of MHC-II on monocytes was measured by flow cytometry. n=3. **(I, K)** B16F10 tumor-bearing mice were administered GSH intraperitoneally twice daily for a total of three days after CTT **(I)**, and the level of ROS in neutrophil was detected using the DCFH-DA **(J)**. The expression level of MHC-II on splenic monocytes was detected on day 5 after CTT **(K)**. *p <0.05, **p <0.01, ***p<0.001, ****p<0.0001. n=4 for each group.

Furthermore, we explored the ability of neutrophils from the CTT group to directly promote the activation of monocytes. Neutrophils were sorted by MACS from B16F10 tumor-bearing mice and CTT treated mice at 12 h (CTT group), and cocultured with neutrophil-free splenocytes for 24 h. In line with the *in vivo* results, neutrophils from the CTT group significantly promoted the expression of MHC-II, CD11c and F4/80 on monocytes compared with those from untreated mice (**Figure 3A**). Similarly, in the 4T1 mouse model, we also found that neutrophils from the CTT group, but not those from the untreated group, increased the levels of MHC-II, CD11c and F4/80 on monocytes. (**Supplementary Figure 7**). These data confirm that the CTT induced systemic neutrophil response at an early stage effectively promoted the activation of monocytes directly. Furthermore, to explore whether the neutrophil-induced activation of monocytes after CTT depends on direct cell-to-cell contact, monocytes isolated from the spleen were treated with neutrophil-conditioned medium. The neutrophil-conditioned medium from the CTT group promoted the activation of monocytes more effectively than that from the untreated group (**Figure 3B**), which indicated that the activation of monocytes promoted by CTT-activated neutrophils was contact-independent.

Activated neutrophils produce ROS via NADPH oxidase (26). GSEA revealed that the ROS biosynthetic process pathway was significantly enriched in neutrophils after CTT compared with untreated mice (**Figure 3C**). Then, ROS in the neutrophils were detected by DCFH-DA fluorescence staining. Compared with other cells, neutrophils presented the highest levels of ROS (**Figure 3D**). Moreover, the level of ROS in neutrophils increased at 6 h and 12 h after CTT (**Figure 3E**). Because ROS can induce M1 macrophage polarization through the MAPK–NFkappaB P65 signaling pathway, we hypothesized that neutrophils promote the expression of MHC-II on monocytes by ROS. Thus, splenic neutrophils from CTT mice were co-cultured with monocytes treated with varying concentrations of GSH, a ROS scavenger. GSH downregulated the expression of MHC-II on monocytes in a dose-dependent manner (**Figure 3F**). Scavenging ROS in the culture medium alone did not affect monocyte activation (**Figure 3G**). However, the increased expression of MHC-II on monocytes induced by neutrophil-conditioned medium was abrogated following the administration of GSH (**Figure 3G**). Importantly, the addition of H_2_O_2_ significantly upregulated the expression of MHC-II on monocytes (**Figure 3H**). To further verify the role of ROS in monocyte activation after CTT, GSH was intraperitoneally administered twice daily for a total of three days to scavenge the ROS of neutrophils (**Figures 3I, J**). Compared with that of CTT alone, the expression of MHC-II on monocytes markedly decreased after GSH admonition (**Figure 3K**). These results suggest that CTT induces ROS production by neutrophils to promote the activation of monocytes.

### 3.3 The CTT induced systemic neutrophil response promoted the effect function of T cells and NK cells

The results of our previous study showed that CTT induced long-lasting systemic antitumor immunity to promote long-term survival in mice (27). To explore whether CTT-activated neutrophils are indispensable for the systemic antitumor immune response, the immune landscape of mice in the late stage (on day 14 after treatment) was analyzed by using flow cytometry. On day 14 after CTT, monocytes in the spleen fully matured with significantly upregulated expression of MHC-II in the spleen (**Supplementary Figures 8A, B**). Meanwhile, the expression of MHC-II and CD11c on monocytes from the lung was increased, while the expression of F4/80 on monocytes from the spleen, blood and lung was downregulated (**Supplementary Figure 8A**). However, after CTT with anti-Ly6G treatment, the expression of MHC-II, CD11c and F4/80 on monocytes in the spleen was significantly decreased compared with that in the CTT group (**Supplementary Figure 8A**). CTT with anti-Ly6G treatment did not affect the expression of MHC-II on macrophages or DCs from the spleen or lung (**Supplementary Figures 8A, B**).

**Figure 4:**
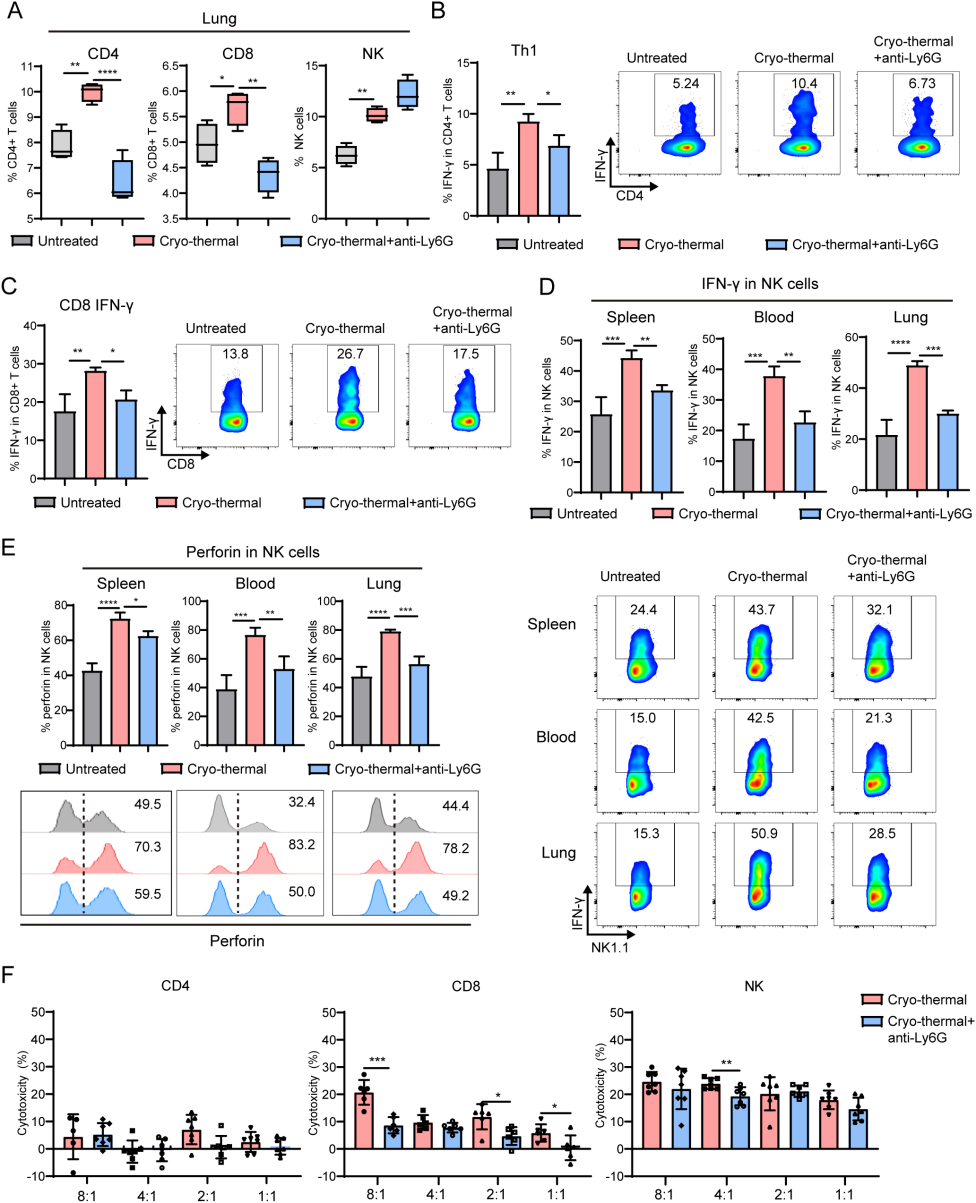
Neutrophils after CTT promoted the effect function of T cells and NK cells. At 12 d after tumor incubation, mice were treated with CTT. Anti-Ly6G antibody was used one day before CTT to deplete the neutrophils. The percentages and cytokines profile of CD4^+^ T-cells, CD8^+^ T-cells and NK cells were measured by flow cytometry 14 d after CTT. **(A)** The percentages of CD4^+^ T-cells, CD8^+^ T-cells and NK cells in lung. **(B)** The percentage of Th1 (IFN-γ^+^) in CD4^+^ T-cells from spleen. **(C)** The expression level of IFN-γ in CD8^+^ T-cells from spleen. **(D, E)** The expression levels of IFN-γ **(D)** and perforin **(E)** in NK cell from spleen, blood and lung. **(F)** Splenic T cell and NK cells from CTT group and untreated mice were co-incubated with calcein-AM-loaded B16F10 cells at a ratio of 8:1, 4:1, 2:1 and 1:1 for 6 h, and the kill rate was calculated by the level of calcein release. *p <0.05, **p <0.01, ***p<0.001, ****p<0.0001. n=4 for each group.

Moreover, on day 14 after CTT, the percentages of CD4^+^ and CD8^+^ T cells and NK cells were sharply increased in the spleen and lung, but did not markedly change in the blood (**Supplementary Figures 9A, B**, **Figure 4A**). However, the increase in CD4^+^ and CD8^+^ T cells in the lung was abolished after neutrophil depletion, which indicated that the activated neutrophils promoted T cells infiltration into the lung after CTT (**Figure 4A**). The cytokine profiles of T cells and NK cells were subsequently analyzed. The results showed that IFN-γ and perforin in CD4^+^ and CD8^+^ T cells and NK cells was comprehensively upregulated in the spleen, blood and lung after CTT (**Figures 4B–E**, **Supplementary Figures 9C–E**), which suggested that CTT induced the activation and cytotoxicity of T cells and NK cells. Although CTT with neutrophil depletion did not affect the proportions of Th2, Th17, TfH and Treg subsets and the expression of IFN-γ and perforin in CD4^+^ and CD8^+^ T-cells in the blood or lungs (**Supplementary Figures 9C–E**), the increase in IFN-γ in splenic CD4^+^ and CD8^+^ T cells after CTT was abolished by neutrophil depletion (**Figures 4B, C**). Most notably, the expression of IFN-γ and perforin in NK cells was dramatically decreased in the spleen, blood and lungs (**Figures 4D, E**). Finally, the cytotoxicity of T-cells and NK cells to B16F10 cells was measured. Compared with that in the CTT group, the cytotoxicity of CD8^+^ T-cells and NK cells was decreased after CTT with neutrophil depletion (**Figure 4F**). CD4^+^ T cells exhibited very low cytotoxicity against B16F10 cells (**Figure 4F**). These findings suggest that the neutrophil response in the early stage promoted the cytotoxicity of CD8^+^ T-cells and NK cells.

### 3.4 Monocytes activated by neutrophils promoted the function of T-cells and NK cells

**Figure 5:**
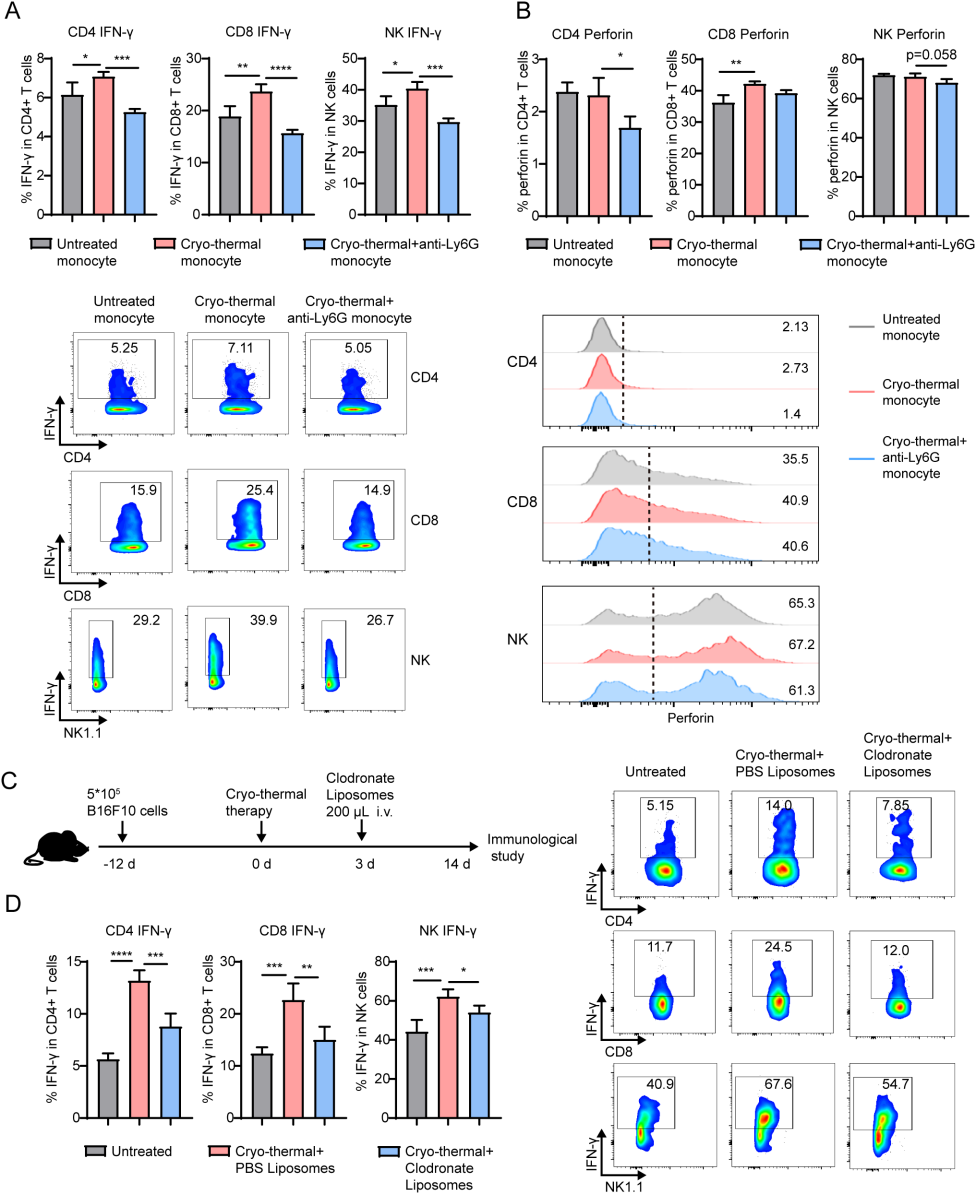
Monocytes activated by neutrophil facilitated the Th1 differentiation and enhanced the cytotoxicity of T-cell and NK cells. Monocytes sorted from untreated mice, CTT mice and CTT with anti-Ly6G mice were cocultured with monocyte-free splenocytes at a ratio of 1:5 for 24h. **(A, B)** The expression of IFN-γ **(A)** and perforin **(B)** in CD4^+^ T-cells, CD8^+^ T-cells and NK cells was measured by flow cytometry. **(C)** Scheme of experiment design. **(D)** Monocytes was depleted by clodronate liposome injection i.v. at 3 d after CTT and the expression of IFN-γ in CD4^+^, CD8^+^ T-cells and NK cells from spleen was measured by flow cytometry. *p <0.05, **p <0.01, ***p<0.001, ****p<0.0001. n=4 for each group.

Because the combination of CTT and neutrophil depletion significantly inhibited the functions of T and NK cells, the underlying mechanisms were addressed. Since the above results indicate that neutrophils have little direct contribution to the differentiation and function of T cells and NK cells at the early stage after CTT, we hypothesized that systemic antitumor immunity is mediated by monocytes, which are activated by neutrophils after CTT. Therefore, monocytes from the untreated, CTT and CTT with anti-Ly6G groups were sorted and coincubated with spleen cells from tumor-bearing mice. Indeed, monocytes from the CTT group, but not from the CTT with anti-Ly6G group, significantly upregulated the expression of IFN-γ in CD4^+^ T-cells, CD8^+^ T-cells and NK cells compared with monocytes from tumor-bearing mice (**Figure 5A**). In addition, monocytes from the CTT with anti-Ly6G group suppressed the expression of perforin in CD4^+^ T-cells and NK cells compared to monocytes from the CTT group (**Figure 5B**). Moreover, monocytes from the CTT with GSH treated group also decreased the expression of IFN-γ in CD4^+^ T-cells, CD8^+^ T-cells and NK cells compared with monocytes from the CTT group (**Supplementary Figure 10A**). To further investigate the effects of neutrophil-activated monocytes on T-cells and NK cells *in vivo*, clodronate liposomes were administered i.v. 3 days after CTT to deplete monocytes (**Figure 5C**). The administration of clodronate liposomes reduced the proportion of monocytes in the blood and spleen from 2.15% to 0.23% and from 0.76% to 0.037% respectively (**Supplementary Figure 10B**). Similar to the results presented above, the levels of IFN-γ and perforin in CD4^+^ and CD8^+^ T-cells and NK cells from the spleen were increased at 14 d after CTT compared with those in untreated mice (**Figure 5C**; **Supplementary Figure 9C**). Although CTT depletion of monocytes did not alter the expression level of perforin in CD4^+^, CD8^+^ T-cells and NK cells, it significantly reduced IFN-γ expression levels in those cells from the spleen (**Supplementary Figure 10B**, **Figure 5D**). These results suggest that the activation of monocytes induced by neutrophils after CTT promots the differentiation of CD4 Th1 cells and the functions of CD8^+^ T-cells and NK cells, thereby orchestrating systemic antitumor immunity.

### 3.5 The neutrophil response enhances the efficacy of CTT and inhibits metastasis

**Figure 6:**
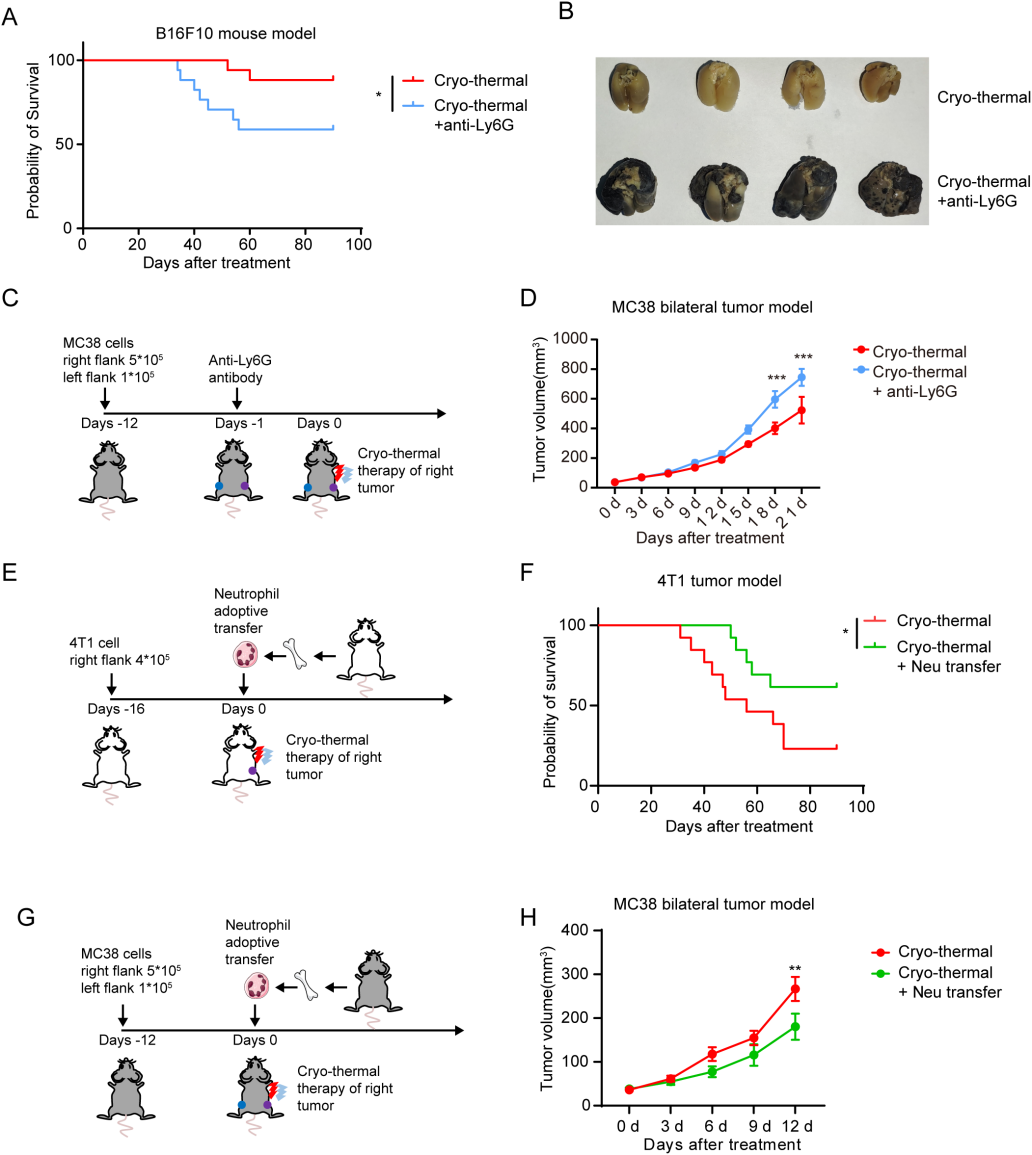
The neutrophil response enhanced the efficacy of CTT. **(A)** Survival curve of B16F10 tumor-bearing mice following treatment with CTT and anti-Ly6G. n=17. **(B)** The images depicted the lungs of mice in the CTT group and in the CTT with anti-Ly6G group. **(C)** Neutrophil depletion strategy for the MC38 bilateral tumor model. **(D)** The right tumors of MC38 bilateral tumor model mice were subjected to CTT, and the volume of left tumors in CTT group and CTT with anti-Ly6G group were monitored. n=6. The results were expressed as the mean ± Standard error of mean (SEM). **(E)** The neutrophil adoptive transfer strategy for 4T1 tumor mouse model. **(F)** Survival curve of 4T1 tumor-bearing mice following CTT or CTT with neutrophil adoptive transfer. n=13. **(G)** The neutrophil adoptive transfer strategy for MC38 bilateral tumor model. **(H)** The right tumors of MC38 bilateral tumor model mice were subjected to CTT, and the volume of left tumors in CTT group and CTT with neutrophil adoptive transfer group were monitored. n=6. The results were expressed as the mean ± Standard error of mean (SEM). *p<0.05, **p<0.01, ***p<0.001.

Our previous studies have shown that CTT induces strong systemic anti-tumor immunity to improve the survival rate and suppress tumor metastasis (21, 27). Given the pivotal role of neutrophils in monocyte activation and the subsequent effector function of T-cells and NK cells after CTT, we sought to investigate whether neutrophils influence the therapeutic efficacy of CTT. Indeed, the depletion of neutrophils significantly reduced the survival of mice after CTT in the B16F10 melanoma model (**Figure 6A**). Concurrently, the depletion of neutrophils resulted in the formation of a considerable number of metastases in the lungs of the mice (**Figure 6B**). To observe the effect of CTT-induced neutrophil response on metastatic tumors, a bilateral MC38 tumor model was used to simulate tumor metastasis. The right-sided tumors of MC38 tumor-bearing mice were subjected to CTT, and the volume of the left-sided tumors were monitored (**Figure 6C**). Compared with CTT alone, the depletion of neutrophils following CTT resulted in a significant increase in the volume of the left tumor (**Figure 6D**). These results suggest that the neutrophil response induced following CTT is essential for the inhibition of tumor metastasis.

We then proceeded to investigate the potential for enhancing the neutrophil response at the time of CTT to contribute to the efficacy of CTT. To this end, the 4T1 breast cancer model, which is characterized by high immunosuppression and spontaneous metastasis, was utilized. Neutrophils isolated from the bone marrow of naïve mice were adoptively transferred into 4T1 tumor-bearing mice, which were then subjected to CTT (**Figure 6E**). Compared with CTT alone, CTT with adoptive neutrophil transfer significantly prolonged the survival of mice and improved the survival rate (**Figure 6F**). Furthermore, CTT with adoptive neutrophil transfer inhibited the growth of left-sided tumors in MC38 bilateral tumor model mice (**Figures 6G, H**). These findings indicate that adoptive neutrophil transfer enhances the efficacy of CTT.

## 4 Discussion

In this study, we proposed that the CTT-induced systemic neutrophil response at an early stage subsequently promoted long-term systemic antitumor immunity. Mechanistically, CTT promoted the recruitment and activation of neutrophils, which promoted the activation of monocytes via ROS. Furthermore, activated monocytes induced by neutrophils after CTT stimulated Th1 cytokine expression in CD4^+^ T-cells and enhanced the effector function of CD8^+^ T-cells and NK cells, thereby orchestrating long-term systemic antitumor immunity. The amplification of the CTT-induced neutrophil response by the combination of CTT with adoptive neutrophil transfer further enhanced the antitumor efficacy in multiple metastatic tumor models, which demonstrated the critical role of neutrophils in CTT-induced antitumor immunity.

Previously, we demonstrated macrophage polarization toward the M1 phenotype on day 5 and fully matured DCs on day 14 after receiving CTT, which ultimately triggered Th1-mediated durable antitumor immunity (4, 28, 29). However, the specific immune cells that promptly respond after CTT had not been identified. In this study, we found that neutrophils were the initial responders after CTT and provoked a complex series of immune cascade responses. Thus, the early emergence of a robust neutrophil response after CTT is needed to stimulate durable antitumor immunity.

Neutrophils are involved in the communication network that forms the basis of immunity, issuing instructions to almost all other immune cells (30). However, very little attention has been given to the crosstalk between neutrophils and monocytes in tumor models. Activated neutrophils induce DC maturation and facilitate the release of cytokines from monocytes and macrophages (31, 32). Moreover, in an influenza infection model, neutrophils can increase the expression of MHC-II and CD11c on monocytes via the secretion of the cytokine EGF (33). In this study, we found that the CTT-induced neutrophil response promoted the expression of MHC-II, F4/80 and CD11c on monocytes in a tumor model. Neutrophils can produce a substantial quantity of ROS in response to various types of stimuli, which play pivotal roles in the response to infection and the regulation of inflammatory processes (34). Moreover, ROS can promote M1-type polarization in macrophages via the ROS-MAPK-NFκB P65 signaling pathway. Our findings indicate that CTT significantly increases the production of ROS by neutrophils. Moreover, the ROS generated by neutrophils are essential for the activation of monocytes. These findings emphasize the vital role of neutrophil-monocyte interactions in the development of CTT-induced durable antitumor immunity and reveal a novel mechanism by which neutrophils facilitate monocyte activation.

Although DCs are considered the most potent APCs, emerging evidence supports a role for monocyte-derived APCs in the priming of naïve or memory T-cells (35, 36). Numerous studies have shown that monocytes can act as APCs, process antigens and present them to T cells via MHC-molecules to activate TCR signaling (35, 37, 38). Moreover, the binding of costimulatory molecules expressed on mature APCs to their ligands provides an additional signal to effectively activate T-cells (39). Inflammatory monocytes are key providers of TNF superfamily costimulatory signals for T-cell activation (40). In the present study, both the RNA-seq and flow cytometry results showed that the systemic neutrophil response was responsible for antigen processing and presentation capacity and the expression of costimulatory molecules in monocytes, which suggested that CTT-induced neutrophils can effectively promote the activation of monocytes. Indeed, the depletion of monocytes after CTT significantly reduced the expression of IFN-γ in T-cells and NK cells. Our study further demonstrated the significant role of monocytes in antitumor immunity. Monocytes have the potential to differentiate into monocyte-derived DCs. In this study, CTT-induced neutrophils promoted the expression of CD11c on monocytes, which suggested that monocytes differentiate toward DCs after activation. However, additional evidence is needed to confirm whether monocytes ultimately differentiate into DCs. Nevertheless, it has been demonstrated that monocytes can retain their own characteristics, thereby performing immune functions without undergoing differentiation into macrophages or DCs (37). In the future, the mechanisms of monocyte differentiation induced by neutrophils following CTT warrant further investigation.

Under the stimulation of tumor-derived factors, neutrophils and monocytes are pathologically activated to form PMN-MDSCs and M-MDSCs, respectively, thus participating in tumor immunosuppression (41). In contrast, upon activation by immunostimulatory agents such as STING agonists, and Dectin-1 ligand, neutrophils and monocytes can be reprogrammed to exert antitumor effects (42–45). Therefore, immune reprogramming of neutrophils and monocytes is a promising cancer therapy that has been recently investigated. The use of combined therapies, such as CD40 agonists, tumor necrosis factor and tumor-binding antibodies drives the neutrophil-mediated eradication of cancer (10). All-trans retinoic acid eliminates immature MDSCs and abrogates MDSC-mediated immunosuppression (46). In this study, we developed a novel thermophysical therapy to target both neutrophils and monocytes that reprogrammed these cells to an antitumor phenotype. Therefore, the results of our study suggest that CTT might represent an ideal tumor immunotherapy strategy for targeting and reprogramming both PMN-MDSCs and M-MDSCs to boost durable antitumor immunity.

However, the process by which CTT induces a neutrophil response appears to be complex. Initial neutrophil recruitment depends on the binding of adhesion molecules on endothelial cells and their ligands on neutrophils, which increases increasing the chance of chemotactic agents binding to ligands on neutrophils (47). Next, neutrophils infiltrate damaged tissue via a gradient of chemotactic agents (48). In our previous study, CTT induced tumor cell necrosis and promoted the release of a large amount of DAMPs including HSP70, HMGB1 and CRT (20, 21), which upregulate the expression of adhesion molecules on endothelial cells to promote neutrophil adhesion (48). At the same time, necrosis of cells may result in the release of mitochondrial contents including formylated peptides and ATP. Formyl peptides can exert a potent chemotactic effect directly on neutrophils (18, 49), while ATP further enhances the response to the preexisting chemoattractant gradient (50). Therefore, collectively, the release of multiple DAMPs after CTT contributes to neutrophil recruitment. The underlying mechanisms are under further study.

In conclusion, the results of this study revealed that CTT induces an early neutrophil response that includes neutrophils, monocytes, T-cells and NK cells to result in durable antitumor immunity. This study demonstrated that CTT could not only be developed as an ideal tumor immunotherapy strategy but also as platform technology to target MDSCs in combination with other immunotherapies in malignant tumors.

## Data Availability

The datasets presented in this study can be found in online repositories. The names of the repository/repositories and accession number(s) can be found below: https://www.ncbi.nlm.nih.gov/geo/, GSE259010.

## References

[1] Khalil DN Smith EL Brentjens RJ Wolchok JD. The future of cancer treatment: immunomodulation, CARs and combination immunotherapy. Nat Rev Clin Oncol. (2016) 13:394. doi: 10.1038/nrclinonc.2016.65 27118494 PMC5558237

[2] Bagchi S Yuan R Engleman EG. Immune checkpoint inhibitors for the treatment of cancer: clinical impact and mechanisms of response and resistance. Annu Rev Pathol-Mech. (2021) 16:223–49. doi: 10.1146/annurev-pathol-042020-042741 33197221

[3] Ginefra P Lorusso G Vannini N. Innate immune cells and their contribution to T-cell-based immunotherapy. Int J Mol Sci. (2020) 21:4441. doi: 10.3390/ijms21124441 32580431 PMC7352556

[4] He K Jia S Lou Y Liu P Xu LX. Cryo-thermal therapy induces macrophage polarization for durable anti-tumor immunity. Cell Death disease. (2019) 10:216. doi: 10.1038/s41419-019-1459-7 30833570 PMC6399266

[5] Jia S Li W Liu P Xu LX. A role of eosinophils in mediating the anti-tumour effect of cryo-thermal treatment. Sci Rep. (2019) 9:13214. doi: 10.1038/s41598-019-49734-5 31519961 PMC6744470

[6] Giese MA Hind LE Huttenlocher A. Neutrophil plasticity in the tumor microenvironment. Blood. (2019) 133:2159–67. doi: 10.1182/blood-2018-11-844548 PMC6524564 30898857

[7] Mantovani A Cassatella MA Costantini C Jaillon S. Neutrophils in the activation and regulation of innate and adaptive immunity. Nat Rev Immunol. (2011) 11:519–31. doi: 10.1038/nri3024 21785456

[8] Hajizadeh F Maleki LA Alexander M Mikhailova MV Masjedi A Ahmadpour M. Tumor-associated neutrophils as new players in immunosuppressive process of the tumor microenvironment in breast cancer. Life Sci. (2021) 264:118699. doi: 10.1016/j.lfs.2020.118699 33137368

[9] Hedrick CC Malanchi I. Neutrophils in cancer: heterogeneous and multifaceted. Nat Rev Immunol. (2022) 22:173–87. doi: 10.1038/s41577-021-00571-6 34230649

[10] Linde IL Prestwood TR Qiu JT Pilarowski G Linde MH Zhang XY. Neutrophil-activating therapy for the treatment of cancer. Cancer Cell. (2023) 41:356–72. doi: 10.1016/j.ccell.2023.01.002 PMC9968410 36706760

[11] Gershkovitz M Caspi Y Fainsod-Levi T Katz B Michaeli J Khawaled S. TRPM2 mediates neutrophil killing of disseminated tumor cells. Cancer Res. (2018) 78:2680–90. doi: 10.1158/0008-5472.Can-17-3614 29490946

[12] Cui C Chakraborty K Tang XA Zhou GL Schoenfelt KQ Becker KM. Neutrophil elastase selectively kills cancer cells and attenuates tumorigenesis. Cell. (2021) 184:3163–77:e21. doi: 10.1016/j.cell.2021.04.016 PMC10712736 33964209

[13] Hirschhorn D Budhu S Kraehenbuehl L Gigoux M Schröder D Chow A. T cell immunotherapies engage neutrophils to eliminate tumor antigen escape variants. Cell. (2023) 186:1432–47. doi: 10.1016/j.cell.2023.03.007 PMC10994488 37001503

[14] Ponzetta A Carriero R Carnevale S Barbagallo M Molgora M Perucchini C. Neutrophils driving unconventional T cells mediate resistance against murine sarcomas and selected human tumors. Cell. (2019) 178:346–60. doi: 10.1016/j.cell.2019.05.047 PMC6630709 31257026

[15] Eruslanov EB Bhojnagarwala PS Quatromoni JG Stephen TL Ranganathan A Deshpande C. Tumor-associated neutrophils stimulate T cell responses in early-stage human lung cancer. J Clin Invest. (2014) 124:5466–80. doi: 10.1172/Jci77053 PMC4348966 25384214

[16] Singhal S Bhojnagarwala PS O’Brien S Moon EK Garfall AL Rao AS. Origin and role of a subset of tumor-associated neutrophils with antigen-presenting cell features in early-stage human lung cancer. Cancer Cell. (2016) 30:120–35. doi: 10.1016/j.ccell.2016.06.001 PMC4945447 27374224

[17] Yang JM Kumar A Vilgelm AE Chen SC Ayers GD Novitskiy SV. Loss of CXCR4 in myeloid cells enhances antitumor immunity and reduces melanoma growth through NK cell and FASL mechanisms. Cancer Immunol Res. (2018) 6:1186–98. doi: 10.1158/2326-6066.Cir-18-0045 PMC6170679 30108045

[18] McDonald B Pittman K Menezes GB Hirota SA Slaba I Waterhouse CCM. Intravascular danger signals guide neutrophils to sites of sterile inflammation. Science. (2010) 330:362–6. doi: 10.1126/science.1195491 20947763

[19] Wang J Hossain M Thanabalasuriar A Gunzer M Meininger C Kubes P. Visualizing the function and fate of neutrophils in sterile injury and repair. Science. (2017) 358:111–5. doi: 10.1126/science.aam9690 28983053

[20] Zhu J Lou Y Liu P Xu LX. Tumor-related HSP70 released after cryo-thermal therapy targeted innate immune initiation in the antitumor immune response. Int J hyperthermia: Off J Eur Soc Hyperthermic Oncology North Am Hyperthermia Group. (2020) 37:843–53. doi: 10.1080/02656736.2020.1788173 32654540

[21] Zhu J Zhang Y Zhang A He K Liu P Xu LX. Cryo-thermal therapy elicits potent anti-tumor immunity by inducing extracellular Hsp70-dependent MDSC differentiation. Sci Rep. (2016) 6:27136. doi: 10.1038/srep27136 27256519 PMC4891716

[22] Wang XM Terasaki PI Rankin GW Chia D Zhong HP Hardy S. A new microcellular cytotoxicity test based on calcein am release. Hum Immunol. (1993) 37:264–70. doi: 10.1016/0198-8859(93)90510-8 8300411

[23] Subramanian A Tamayo P Mootha VK Mukherjee S Ebert BL Gillette MA. Gene set enrichment analysis: A knowledge-based approach for interpreting genome-wide expression profiles. Proc Natl Acad Sci United States America. (2005) 102:15545–50. doi: 10.1073/pnas.0506580102 PMC1239896 16199517

[24] Srivastava MK Sinha P Clements VK Rodriguez P Ostrand-Rosenberg S. Myeloid-derived suppressor cells inhibit T-cell activation by depleting cystine and cysteine. Cancer Res. (2010) 70:68–77. doi: 10.1158/0008-5472.Can-09-2587 20028852 PMC2805057

[25] Jakubzick CV Randolph GJ Henson PM. Monocyte differentiation and antigen-presenting functions. Nat Rev Immunol. (2017) 17:349–62. doi: 10.1038/nri.2017.28 28436425

[26] El-Benna J Hurtado-Nedelec M Marzaioli V Marie JC Gougerot-Pocidalo MA Dang PMC. Priming of the neutrophil respiratory burst: role in host defense and inflammation. Immunol Rev. (2016) 273:180–93. doi: 10.1111/imr.12447 27558335

[27] He K Liu P Xu LX. The cryo-thermal therapy eradicated melanoma in mice by eliciting CD4 T-cell-mediated antitumor memory immune response. Cell Death disease. (2017) 8:e2703. doi: 10.1038/cddis.2017.125 28333145 PMC5386530

[28] He K Liu P Xu LX. The cryo-thermal therapy eradicated melanoma in mice by eliciting CD4(+) T-cell-mediated antitumor memory immune response. Cell Death disease. (2017) 8:e2703. doi: 10.1038/cddis.2017.125 28333145 PMC5386530

[29] Peng P Lou Y Wang J Wang S Liu P Xu LX. Th1-dominant CD4(+) T cells orchestrate endogenous systematic antitumor immune memory after cryo-thermal therapy. Front Immunol. (2022) 13:944115. doi: 10.3389/fimmu.2022.944115 35874660 PMC9304863

[30] Amulic B Cazalet C Hayes GL Metzler KD Zychlinsky A. Neutrophil function: from mechanisms to disease. Annu Rev Immunol. (2012) 30:459–89. doi: 10.1146/annurev-immunol-020711-074942 22224774

[31] van Gisbergen KPJM Sanchez-Hernandez M Geijtenbeek TBH van Kooyk Y. Neutrophils mediate immune modulation of dendritic cells through glycosylation-dependent interactions between Mac-1 and DC-SIGN. J Exp Med. (2005) 201:1281–92. doi: 10.1084/jem.20041276 PMC2213143 15837813

[32] Soehnlein O Weber C Lindbom L. Neutrophil granule proteins tune monocytic cell function. Trends Immunol. (2009) 30:546–56. doi: 10.1016/j.it.2009.06.006 19699683

[33] Lim K Kim TH Trzeciak A Amitrano AM Reilly EC Prizant H. *In situ* neutrophil efferocytosis shapes T cell immunity to influenza infection. Nat Immunol. (2020) 21:1046–57. doi: 10.1038/s41590-020-0746-x PMC7791396 32747818

[34] Dahlgren C Karlsson A Bylund J. Intracellular neutrophil oxidants: from laboratory curiosity to clinical reality. J Immunol. (2019) 202:3127–34. doi: 10.4049/jimmunol.1900235 31109945

[35] Adams SF Grimm AJ Chiang CLL Mookerjee A Flies D Jean S. Rapid tumor vaccine using Toll-like receptor-activated ovarian cancer ascites monocytes. J Immunotherapy Cancer. (2020) 8:e000875. doi: 10.1136/jitc-2020-000875 PMC7430560 32817208

[36] Dunbar PR Cartwright EK Wein AN Tsukamoto T Li ZRT Kumar N. Pulmonary monocytes interact with effector T cells in the lung tissue to drive T differentiation following viral infection. Mucosal Immunol. (2020) 13:161–71. doi: 10.1038/s41385-019-0224-7 PMC6917844 31723250

[37] Jakubzick C Gautier EL Gibbings SL Sojka DK Schlitzer A Johnson TE. Minimal differentiation of classical monocytes as they survey steady-state tissues and transport antigen to lymph nodes. Immunity. (2013) 39:599–610. doi: 10.1016/j.immuni.2013.08.007 24012416 PMC3820017

[38] Kim TS Braciale TJ. Respiratory dendritic cell subsets differ in their capacity to support the induction of virus-specific cytotoxic CD8(+) T cell responses. PloS One. (2009) 4:e4204. doi: 10.1371/journal.pone.0004204 19145246 PMC2615220

[39] Chen LP Flies DB. Molecular mechanisms of T cell co-stimulation and co-inhibition. Nat Rev Immunol. (2013) 13:227–42. doi: 10.1038/nri3405 PMC3786574 23470321

[40] Chu KL Batista NV Girard M Watts TH. Monocyte-derived cells in tissue-resident memory T cell formation. J Immunol. (2020) 204:477–85. doi: 10.4049/jimmunol.1901046 31964721

[41] Grover A Sanseviero E Timosenko E Gabrilovich DI. Myeloid-derived suppressor cells: A propitious road to clinic. Cancer Discovery. (2021) 11:2693–706. doi: 10.1158/2159-8290.Cd-21-0764 34635571

[42] Nagata M Kosaka A Yajima Y Yasuda S Ohara M Ohara K. A critical role of STING-triggered tumor-migrating neutrophils for anti-tumor effect of intratumoral cGAMP treatment. Cancer Immunol Immun. (2021) 70:2301–12. doi: 10.1007/s00262-021-02864-0 PMC10992389 33507344

[43] Lam KC Araya RE Huang A Chen QY Di Modica M Rodrigues RR. Microbiota triggers STING-type I IFN-dependent monocyte reprogramming of the tumor microenvironment. Cell. (2021) 184:5338–56. doi: 10.1016/j.cell.2021.09.019 PMC8650838 34624222

[44] Humbert M Guery L Brighouse D Lemeille S Hugues S. Intratumoral CpG-B Promotes Antitumoral Neutrophil, cDC, and T-cell Cooperation without Reprograming Tolerogenic pDC. Cancer Res. (2018) 78:3280–92. doi: 10.1158/0008-5472.Can-17-2549 29588348

[45] Kalafati L Kourtzelis I Schulte-Schrepping J Li XF Hatzioannou A Grinenko T. Innate immune training of granulopoiesis promotes anti-tumor activity. Cell. (2020) 183:771–+. doi: 10.1016/j.cell.2020.09.058 PMC7599076 33125892

[46] Wu YZ Yi M Niu MK Mei Q Wu KM. Myeloid-derived suppressor cells: an emerging target for anticancer immunotherapy. Mol Cancer. (2022) 21:184. doi: 10.1186/s12943-022-01657-y 36163047 PMC9513992

[47] Liew PX Kubes P. The neutrophil’s role during health and disease. Physiol Rev. (2019) 99:1223–48. doi: 10.1152/physrev.00012.2018 30758246

[48] Pittman K Kubes P. Damage-associated molecular patterns control neutrophil recruitment. J innate immunity. (2013) 5:315–23. doi: 10.1159/000347132 PMC6741494 23486162

[49] Carp H. Mitochondrial N-formylmethionyl proteins as chemoattractants for neutrophils. J Exp Med. (1982) 155:264–75. doi: 10.1084/jem.155.1.264 PMC2186576 6274994

[50] Chen Y Corriden R Inoue Y Yip L Hashiguchi N Zinkernagel A. ATP release guides neutrophil chemotaxis via P2Y2 and A3 receptors. Science. (2006) 314:1792–5. doi: 10.1126/science.1132559 17170310

