# Supplementary material for: Neutrophils promote the activation of monocytes via ROS to boost systemic antitumor immunity after cryo-thermal therapy
Source: Front Immunol. 2024 Nov 1;15:1445513. doi: 10.3389/fimmu.2024.1445513 (PMC11563809; doi:10.3389/fimmu.2024.1445513)
Supplement: Supplementary file 1 [file DataSheet1.pdf]

Supplementary Material

Neutrophils promote the activation of monocytes via ROS to boost systemic antitumor immunity after cryo-thermal therapy

Shicheng Wang1, Zelu Zhang1, Junjun Wang1, Yue Lou1, Yongxin Zhu1, Jiaqi You1, Ping Liu1* and Lisa X Xu1*

[1]School of Biomedical Engineering and Med-X Research Institute, Shanghai Jiao Tong University, Shanghai, China.

*** Correspondence:**
Lisa X Xu: lisaxu@sjtu.edu.cn; Ping Liu: pingliu@sjtu.edu.cn

1 Supplementary Figures and Tables

1.1 Supplementary Figures

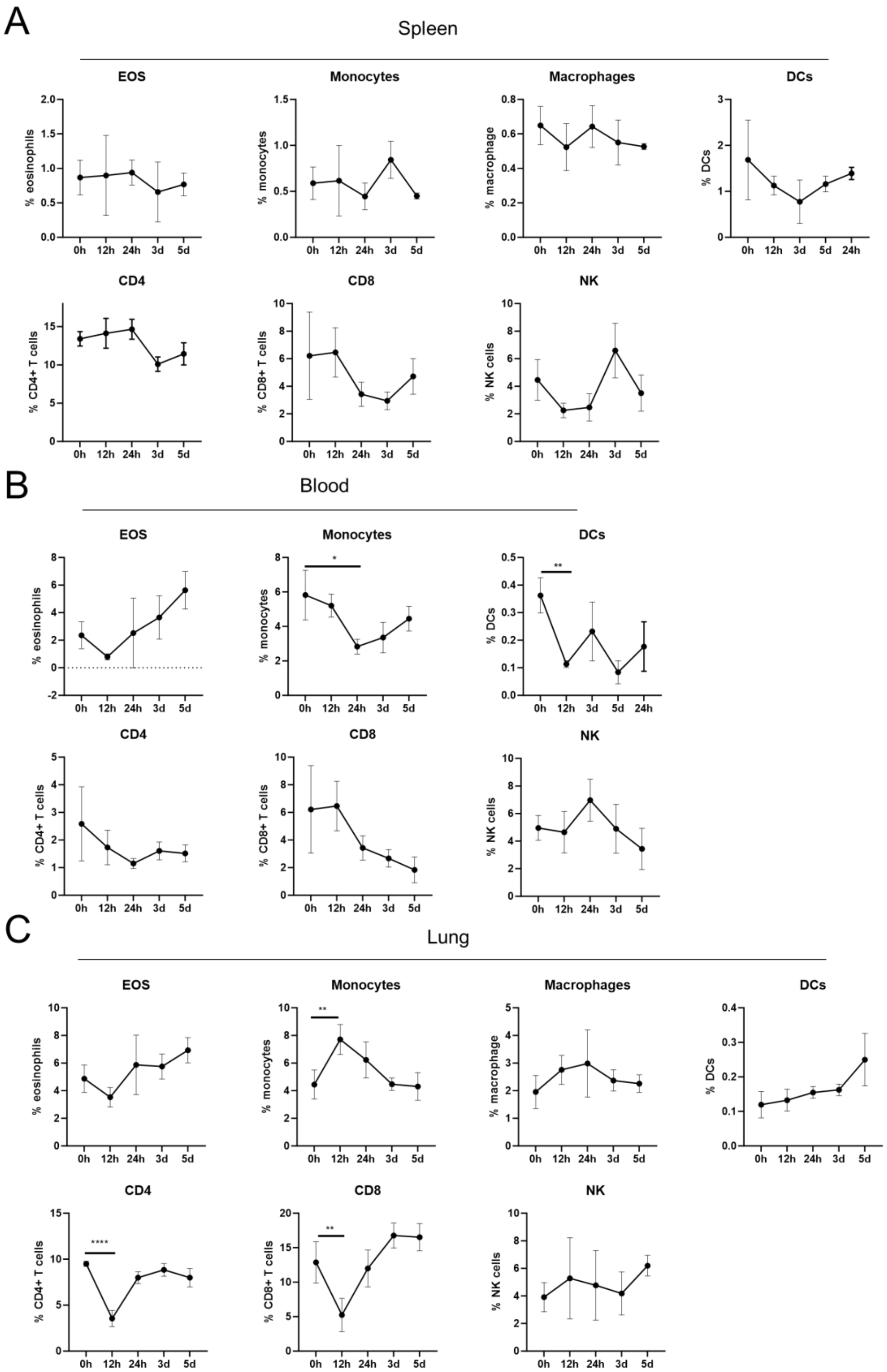
A
Spleen
EOS
Monocytes
Macrophages
DCs
% eosinophils
% monocytes
% macrophage
% DCs
CD4
CD8
NK
% CD4+ T cells
% CD8+ T cells
% NK cells
0h 12h 24h 3d 5d
0h 12h 3d 5d 24h
B
Blood
EOS
Monocytes
DCs
% eosinophils
% monocytes
% DCs
CD4
CD8
NK
% CD4+ T cells
% CD8+ T cells
% NK cells
C
Lung
EOS
Monocytes
Macrophages
DCs
% eosinophils
% monocytes
% macrophage
% DCs
CD4
CD8
NK
% CD4+ T cells
% CD8+ T cells
% NK cells

Supplementary Figure 1. Dynamics of immune cells after CTT. (A-C) The percentage of eosinophils, monocytes, macrophages, DCs, $CD4^+$ T cells, $CD8^+$ T cells and NK cells in spleen, blood and lung in 12 h, 24 h, 3 d and 5 d after CTT was detected by flow cytometry in the B16F10 tumor models. *p <0.05, **p <0.01, ****p<0.0001.. n=4 for each group.

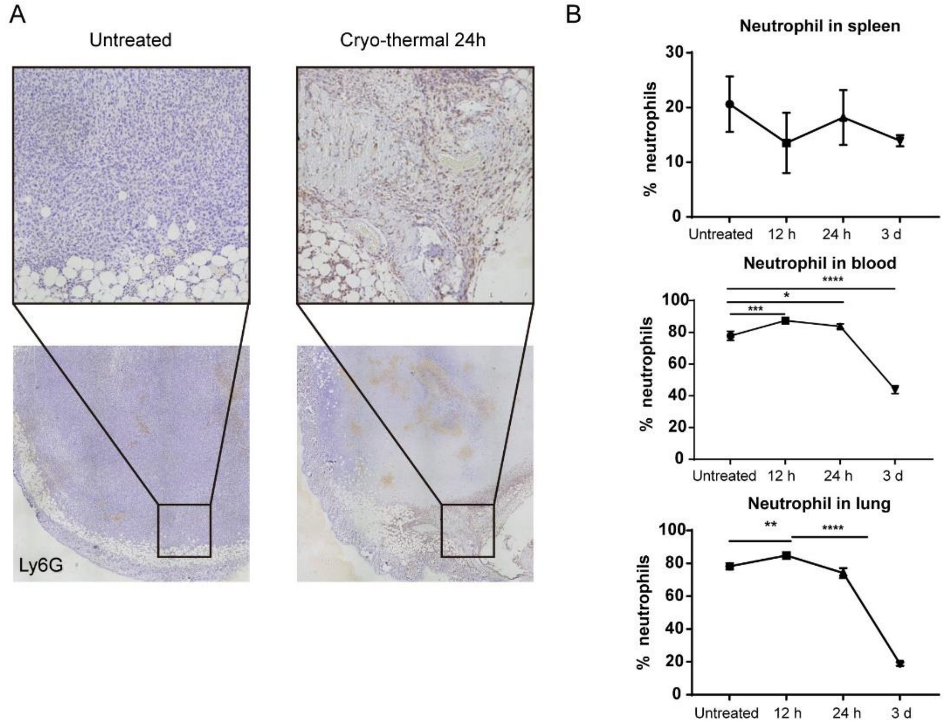

Supplementary Figure 2. CTT induced a rapid neutrophil response in a 4T1 breast cancer mouse model. (A) Representative immunohistochemical staining images of neutrophil ($Ly6G^+$) infiltration in tumor of tumor-bearing mice and CTT mice at 24 h after CTT in the 4T1 tumor model. (B) The percentage of neutrophils in spleen, blood and lung in 12 h, 24 h and 3 d after CTT was detected by flow cytometry in the 4T1 tumor models. *p <0.05, **p <0.01, ***p<0.001, ****p<0.0001. n=4 for each group.

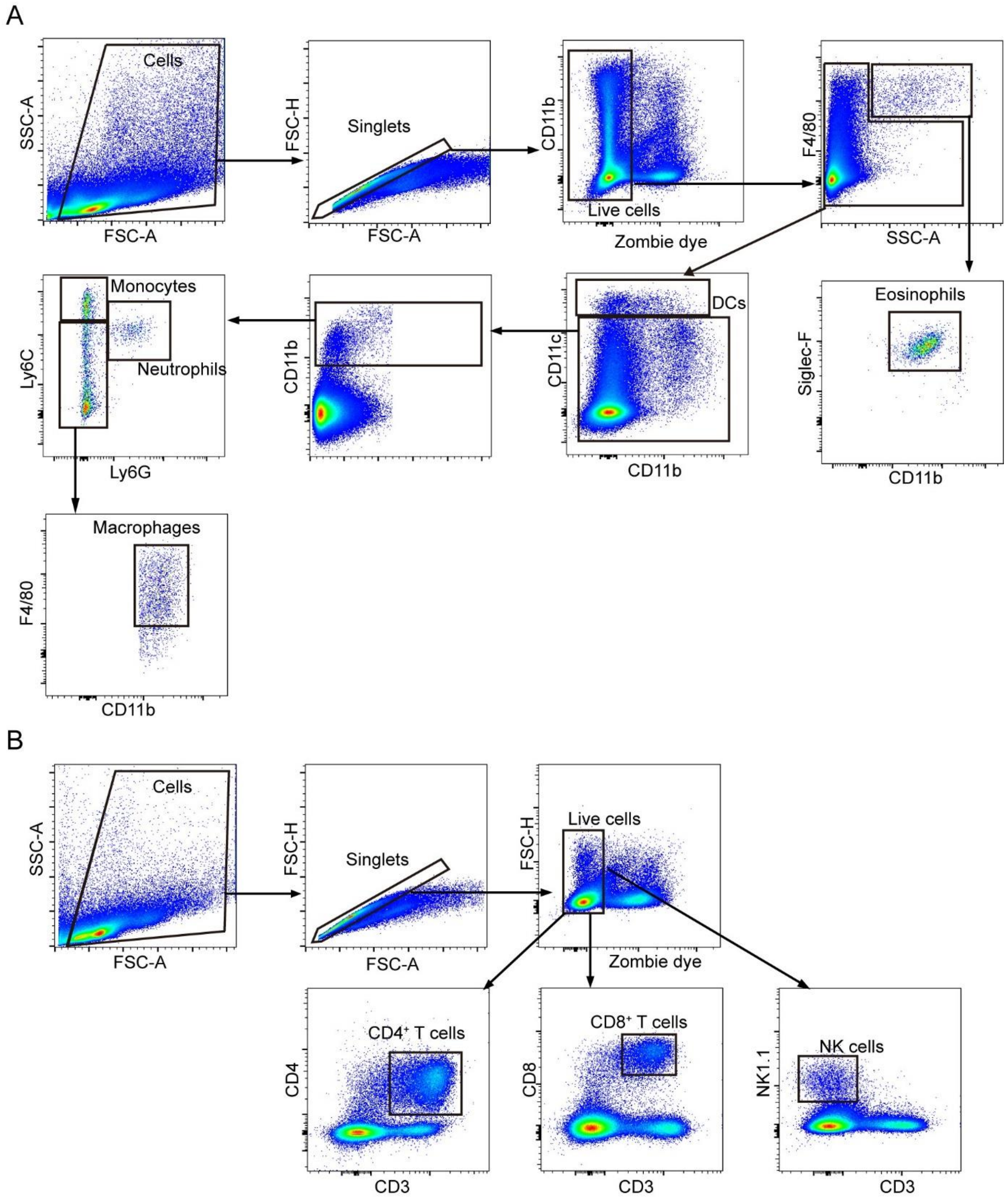

Supplementary Figure 3. The gating strategy of immune cells.

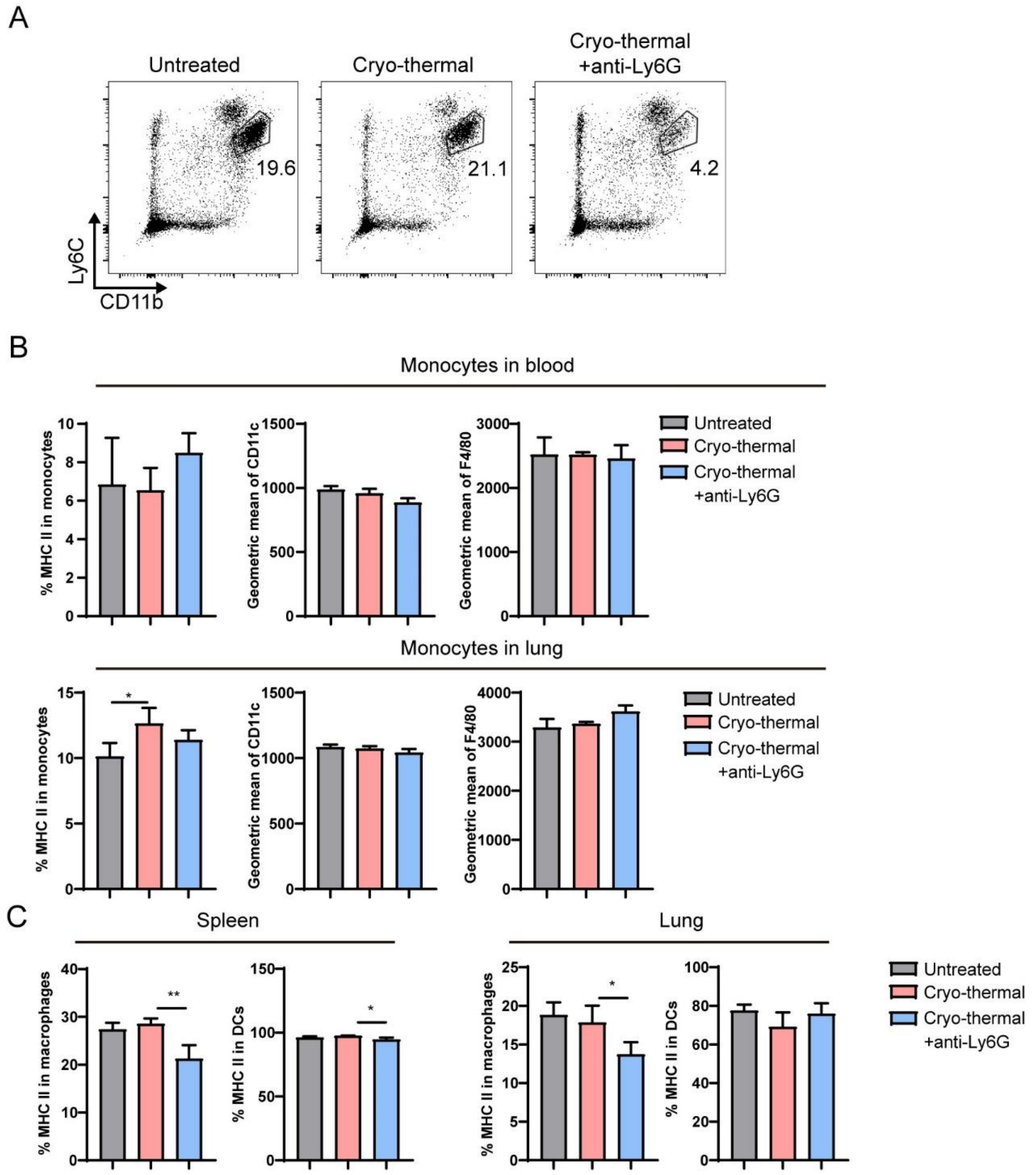

Supplementary Figure 4. Maturation of monocytes, macrophages and DCs in the spleen, blood and lungs. (A) Determination of neutrophil depletion efficiency using flow cytometry. (B-C) The expression of MHC II

in monocytes (B), macrophages (C) and DCs (B) in blood, spleen and lung on day 5 after CTT was detected by flow cytometry. *p <0.05, **p <0.01. n=4 for each group.

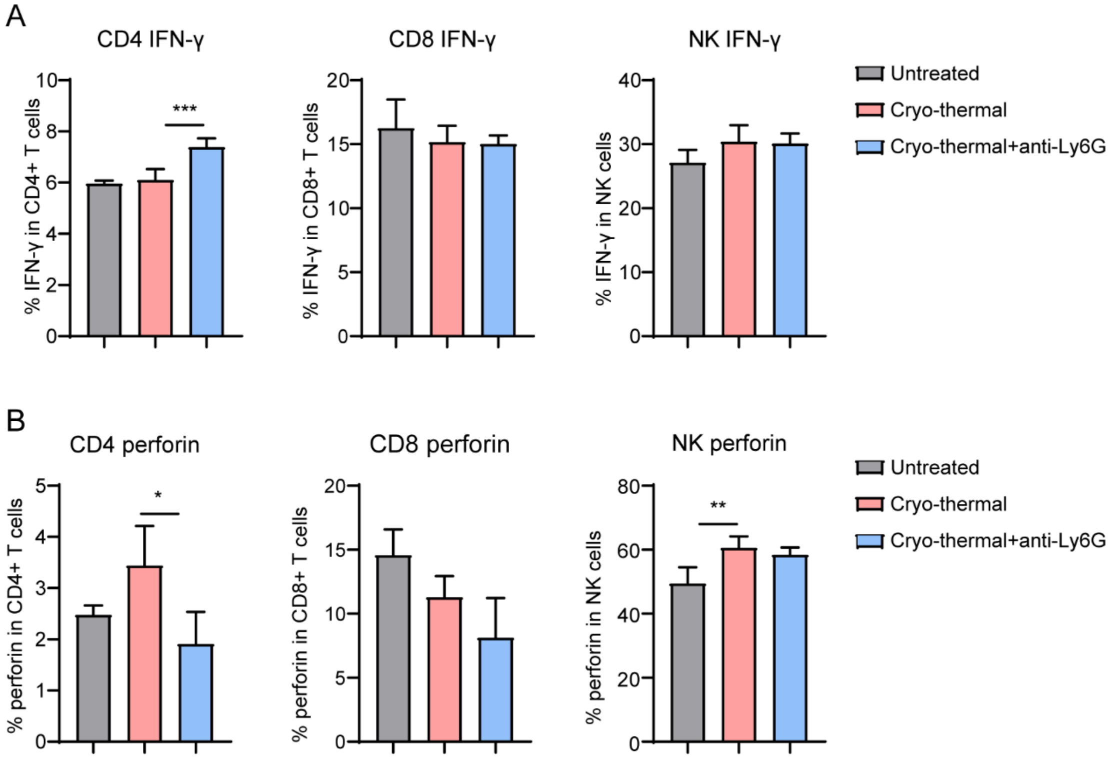

Supplementary Figure 5. The differentiation and function of T-cells and NK cells in spleen 5 d after CTT. At 12 d after tumor incubation, mice were treated with CTT. Anti-Ly6G antibody was used one day before

CTT to deplete the neutrophils. (A-B) The expression of IFN-γ (A) and perforin (B) in $CD4^+$ T-cells, $CD8^+$ T-cells and NK cells was measured by flow cytometry. *p <0.05, **p <0.01, ***p<0.001. n=4 for each group.

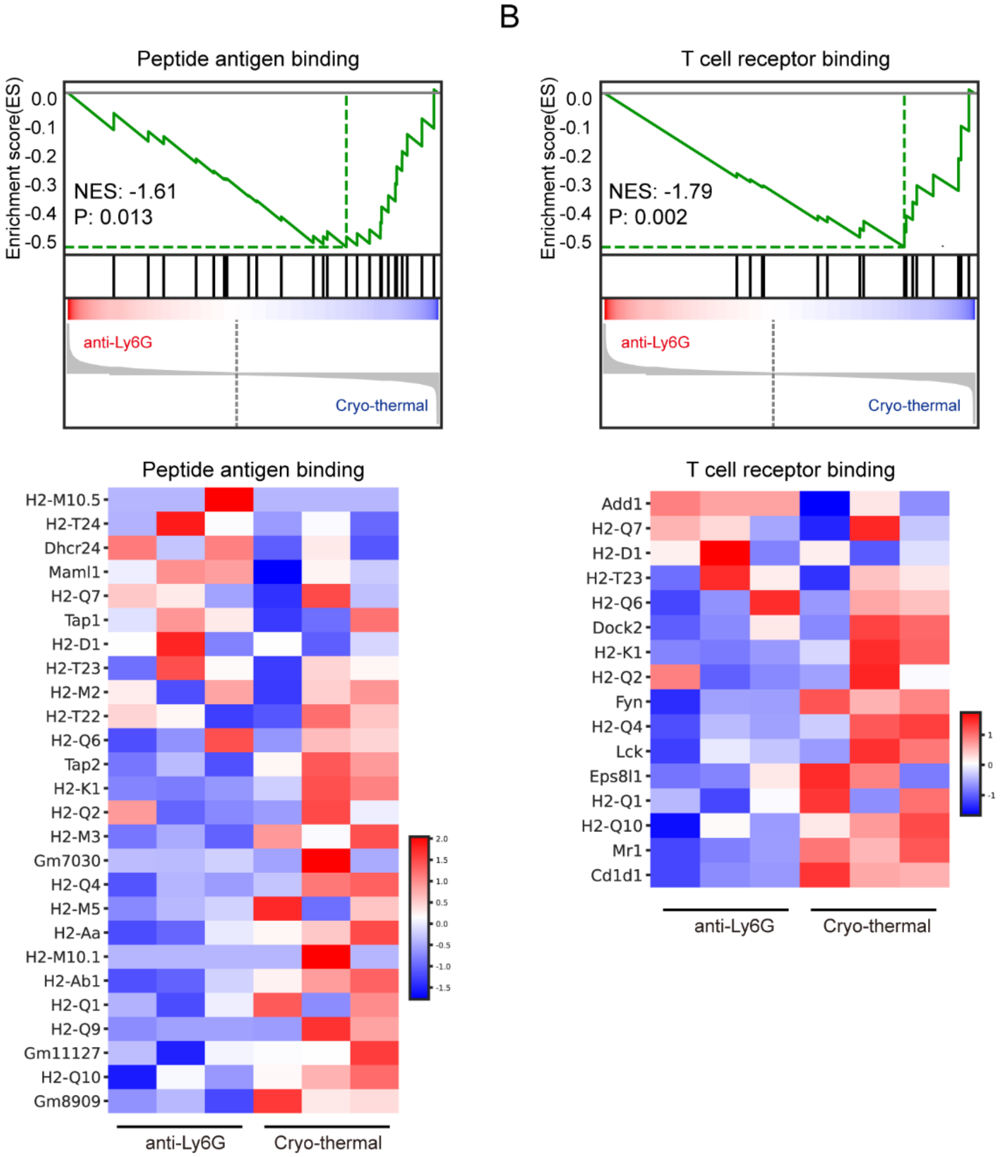

Supplementary Figure 6. Monocytes from CTT and anti-Ly6G group was sorted by MACS and analyzed by mRNA-seq. (A-B) peptide antigen binding (GO:0042605) and T-cell receptor binding (GO:0042608) pathway was analyze by GSEA.

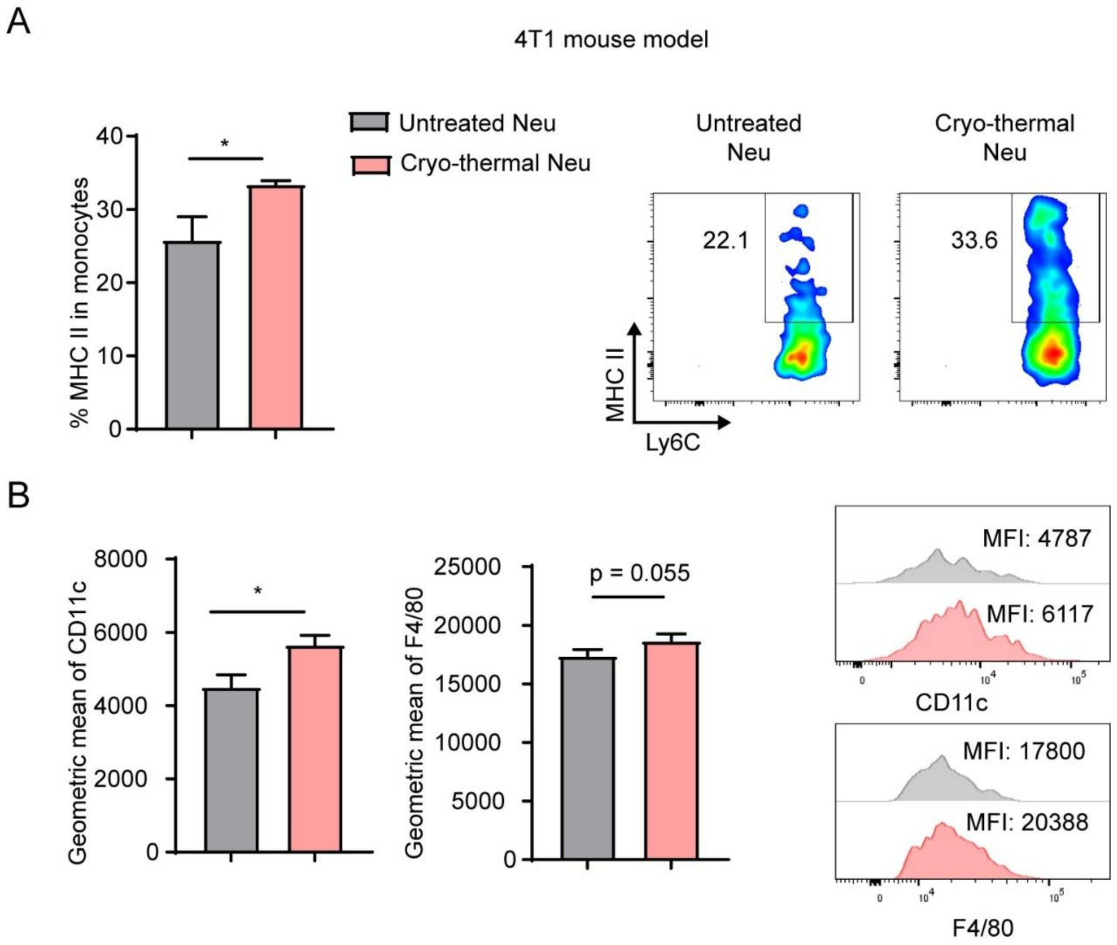

Supplementary Figure 7. Neutrophil promoted the activation of monocyte in 4T1 tumor model. (A-B) Neutrophils sorted from untreated mice and CTT mice in 4T1 tumor models were cocultured with neutrophil-

free splenocytes at a ratio of 1:5 for 24 h. The expression of MHC II, CD11c and F4/80 on monocytes was detected by flow cytometry.

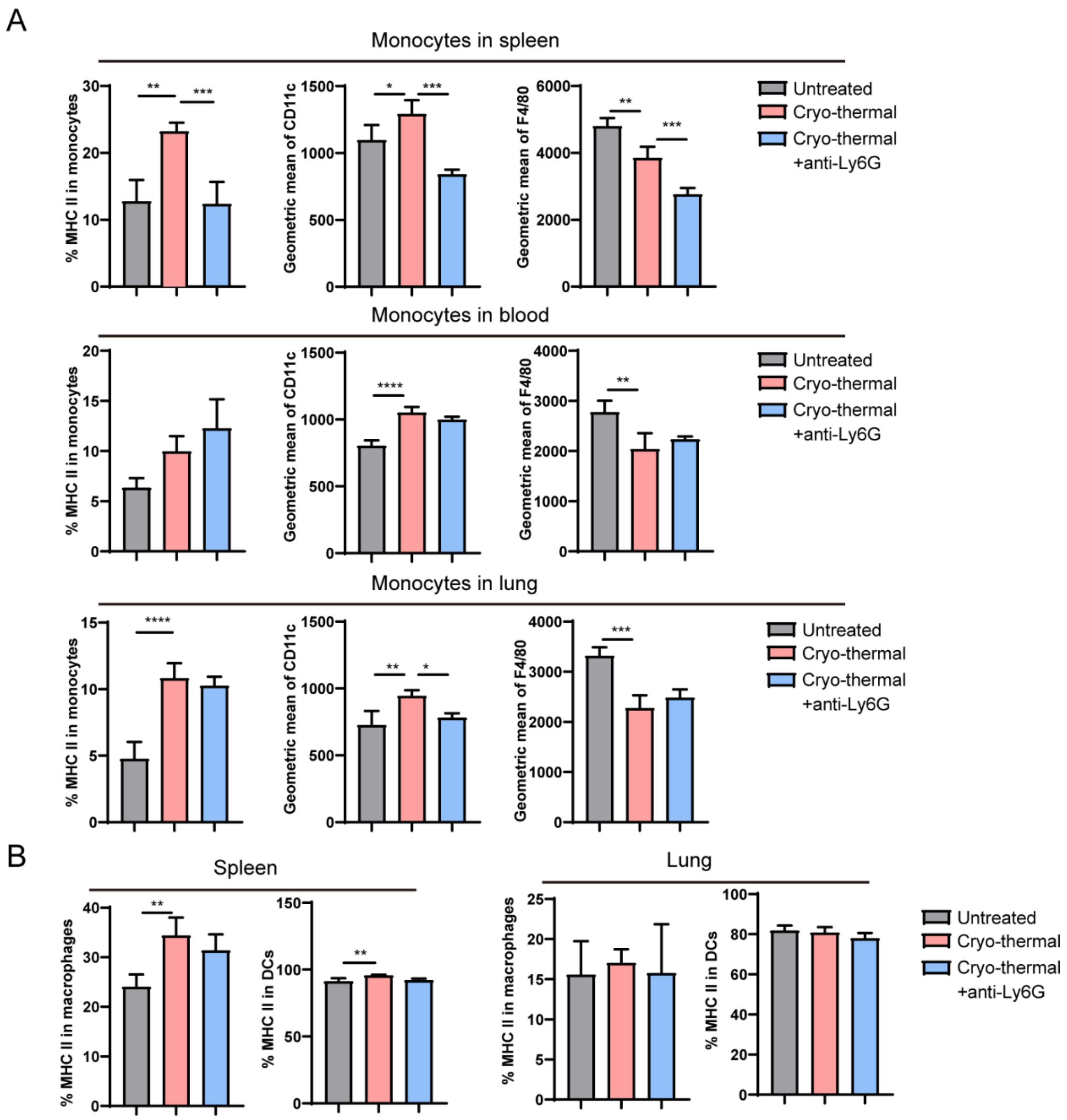

Supplementary Figure 8. Neutrophils induced by CTT promoted the monocyte maturation. At 12 d after tumor incubation, mice were treated with CTT. Anti-Ly6G antibody was used one day before CTT to deplete the neutrophils. The expression levels of MHC II on monocytes, macrophages and DCs were measured by flow cytometry 14 d after CTT. (A) The expression levels of MHC II on monocytes from spleen, blood and

lung. (B-C) The expression levels of MHC II on macrophages (B) and DCs (C) from spleen and lung. *p <0.05, **p <0.01, ***p<0.001. n=4 for each group.

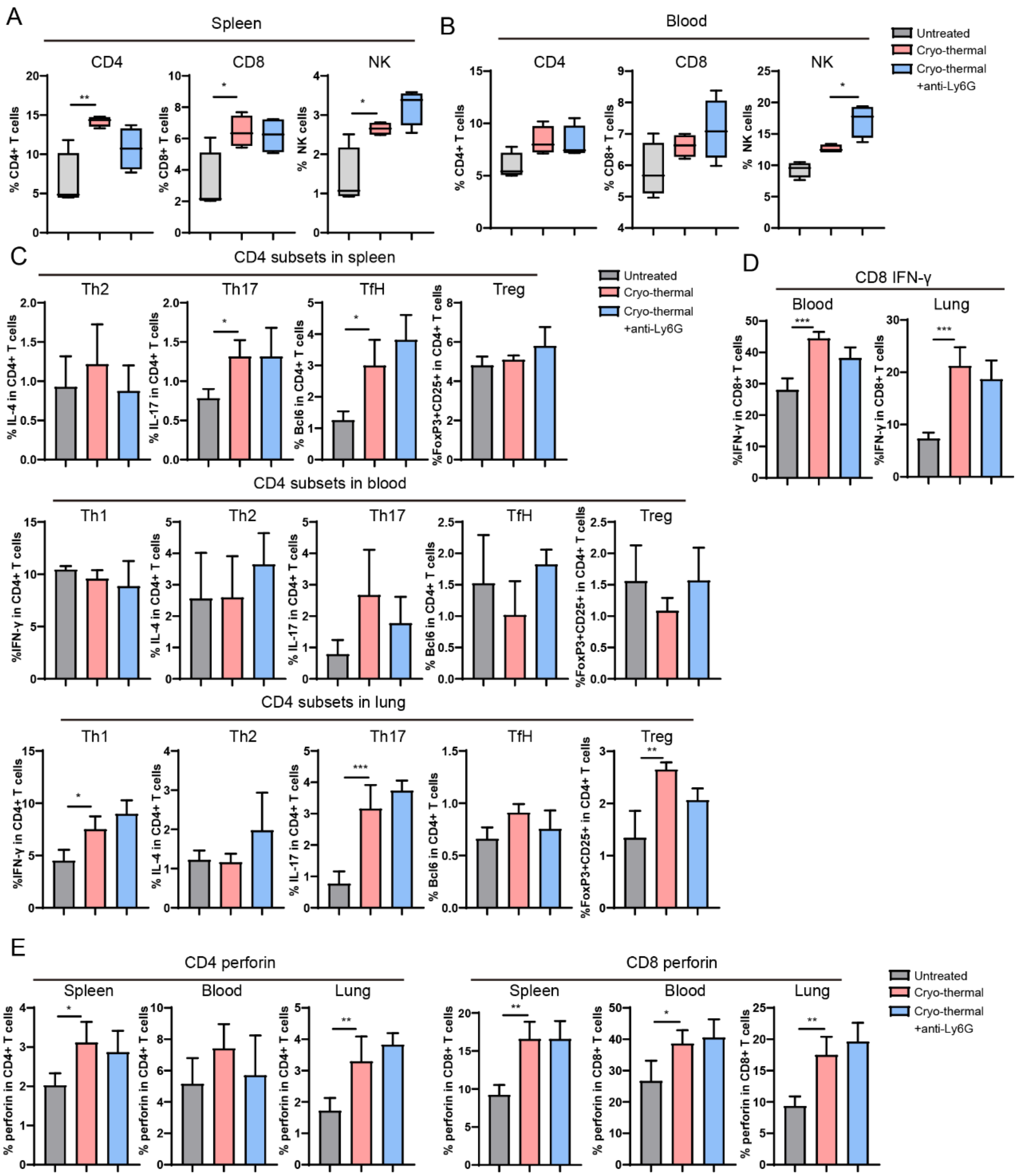

Supplementary Figure 9. Neutrophils after CTT was essential for the systemic anti-tumor immunity. At 12 d after tumor incubation, mice were treated with CTT. Anti-Ly6G antibody was used one day before CTT to deplete the neutrophils. The percentages, subsets and cytokines profile of $CD4^+$ and $CD8^+$ T-cells were measured by flow cytometry 14 d after CTT. (A-B) The percentages of $CD4^+$ T-cells, $CD8^+$ T-cells and NK cells in spleen and blood. (C) The percentages of Th1 (IFN-γ^+), Th2 (IL-4^+), Th17 (IL-17^+), TfH ($Bcl6^+$) and Tregs ($CD25^+FoxP3^+$) in $CD4^+$ T-cells from spleen, blood and lung. (D) The expression level of IFN-γ in

CD8$^+$ T-cells from blood and lung. (E) The expression level of perforin in CD4$^+$ and CD8$^+$ T-cells from spleen, blood and lung. *p <0.05, **p <0.01, ***p<0.001. n=4 for each group.

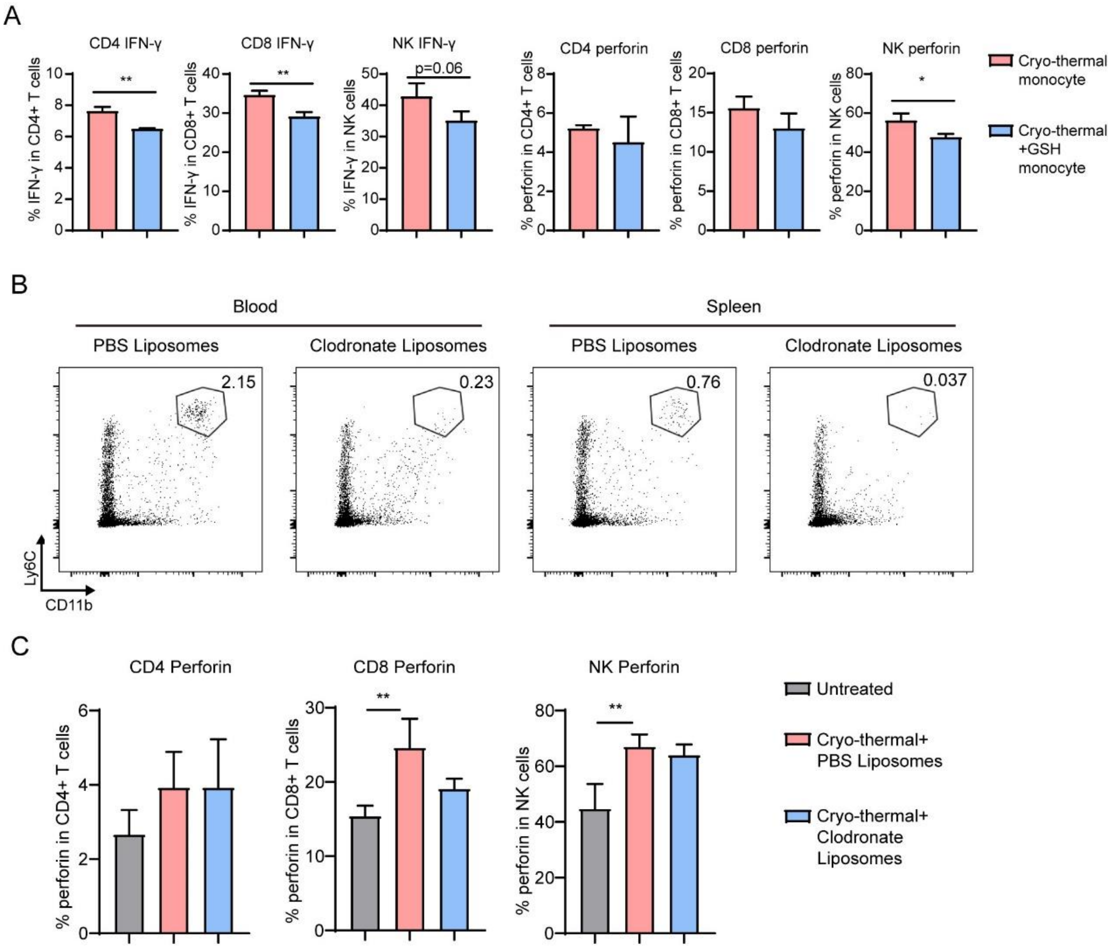

Supplementary Figure 10. Depletion efficiency of monocytes and expression of perforin in T-cells and NK cells. (A) B16F10 tumor-bearing mice were administered GSH intraperitoneally after CTT. Monocytes were sorted and cocultured with monocyte-free splenocytes at a ratio of 1:5 for 24 h. The expression of IFN-γand perforin in CD4+ T-cells, CD8+ T-cells and NK cells was measured by flow cytometry. (B-C) Mice were administrated with chlorophosphate liposome or PBS liposome at 3 d after CTT, the percentages of monocyte in blood and spleen (B) as well as the expression of perforin in T-cells and NK cells (C) were measured by flow cytometry. **p <0.01. n=4 for each group.

1.2 Supplementary Tables

Table S1 Antibodies used in this study.

Antibodies	Company	Clone
Pacific blue-CD11b	BioLegend	M1/70
PE-CD11c	BioLegend	N418
PerCP-Cy5.5-IA/IE	BioLegend	M5/114.15.2
APC-F4/80	BioLegend	BM8
FITC-Ly6C	BioLegend	HK1.4
PE/Cy7-Ly6G	BioLegend	1A8
PerCP-Cy5.5-CD3	BioLegend	145-2411

APC/Cy7-CD4	BioLegend	RM4-5
FITC-NK1.1	BioLegend	PK136
Pacific blue-CD8	BioLegend	53-6.7
PE/Dazzle 594-IFN-γ	BioLegend	XMG1.2
BV421-IL4	BioLegend	11B11
PE-IL-17	BioLegend	TC11-18H10.1
AF647-Granzyme	BioLegend	GB11
PE-Perforin	BioLegend	S16009A
PE/Cy7-CD25	BioLegend	3C7
PE-FoxP3	BioLegend	MF-14
BV421-Bcl6	BD bioscience	K112-91
PE-Ly6G	BioLegend	1A8
PE-CCR2	BioLegend	SA203G11
PE-SiglecF	BD bioscience	E50-2440
